# Dodder-transmitted mobile systemic signals activate a salt-stress response characterized by a transcriptome change in *Citrus sinensis*

**DOI:** 10.3389/fpls.2022.986365

**Published:** 2022-08-09

**Authors:** Shuo Duan, Zhou Xu, Xin-Yu Li, Ping Liao, Hong-Kun Qin, Ya-Ping Mao, Wen-Shan Dai, Hai-Jie Ma, Min-Li Bao

**Affiliations:** ^1^China-USA Citrus Huanglongbing Joint Laboratory, National Navel Orange Engineering Research Center, Gannan Normal University, Ganzhou, China; ^2^College of Horticulture Science, Zhejiang A&F University, Hangzhou, Zhejiang, China

**Keywords:** mobile systemic signals, salt-stress, dodder (*Cuscuta chinensis*), dodder-transmitted, transcriptome change

## Abstract

Citrus is an essential horticultural fruit whose yield and quality are affected by salinity all over the world. The recognition and adaptive regulation of citrus against salt stress are important areas for cultivar improvement, but the vascular system signal transduction mechanism of the plant response to salt stress remains elusive. In this study, we constructed a dodder (*Cuscuta spp.*) linked Hamlin sweet orange (*Citrus sinensis*) plant community in which deliver a vascular signal through the dodder in response to salt stress. RNA-seq technology was used to analyze the gene expression profile of citrus leaves after salt treatment. The results showed that a vascular signal was transmitted to a dodder-linked host plant, triggering a transcriptional response to salt stress. However, the phenotypic and transudative ability of the dodder changed after 24 h. The salt treatment group (Group S) and the dodder-linked group (Group D) respectively contained 1,472 and 557 differentially expressed genes (DEGs). 454 of which were common to both groups. The results of our analysis revealed that the gene expression categories in Group D represented a highly consistent trend compared to the group S plants, indicating that the dodder-bridged vascular signals activated the stress-response of citrus leaves for transcriptomic reconfiguration. The KEGG pathway database and an analysis of key drivers revealed that phenylpropanoid biosynthesis, photosynthesis-antenna proteins, starch and sucrose metabolism, plant hormone signal transduction, circadian rhythm, and MAPK signaling pathways were significantly enriched as the critical genes during salt stress. A systemic signal in the dodder-bridged host significantly regulated abiotic stress-related secondary metabolic pathways, including those for phenylpropanoids, lignin, and lignans. The physiological indexes of photosynthetic intensity, respiration, and attractiveness among communities supported the transcriptional changes. Thus, our results indicate that salt stress-induced vascular system signals can be transmitted through the vascular system of a dodder linking citrus plants, revealing the genetic regulation and physiological changes of citrus leaves responding to plant stress signal transmission.

## Introduction

A abiotic and biotic stresses including drought, low temperature, diseases, pests, and salinity, significantly affect plants during their lifespan (Golldack et al., 2014; Pandey et al., 2017; Zhang et al., 2022). Salinity is one of the most prevalent abiotic stressors that threatens agricultural production. Plants subjected to salt stress can suffer restricted growth due to reduced photosynthesis and damaged cellular structures (Chaves et al., 2009). Chronic salt stress is widespread and decreases the growth and yield of all crops.

Citrus is the most economically important horticultural type of fruit worldwide. However, salt stress adversely affects its yield and fruit quality. Salt induces osmotic stress and ionic toxicity in plants, resulting in stomatal closure, which in turn reduces photosynthesis and transpiration (Arif et al., 2020). Salt stress can also cause nutritional disorders, oxidative stress, membrane effects and other secondary effects such as tissue disorder, and metabolic imbalance; these physiological changes often lead to plant growth inhibition and even death. Thus, improving the salt tolerance of citrus can reduce economic losses and increase the cultivation area, including locations with saline-alkaline soil. The plant plasma membrane is affected by salinity, because the lipid bilayer becomes more fluid, consequently inducing a Ca^2+^ influx into the cytoplasm (Mahajan and Tuteja, 2005). Orderly amplification by Ca^2+^ signaling cascades (e.g., CMLs, CAMs, and CBLs) and MAPK cascades further regulates the expression of a series of salt stress response genes (Xie et al., 2017). Experimental data show that salinity can negatively affect critical plant cellular pathways, such as photosynthesis (Chaves et al., 2009), plant hormone signal transduction, MAPK signaling, circadian rhythm, etc.

Salt hypersensitivity (SOS) genes, reactive oxygen species (ROS), WRKYs, glutathione peroxidase (Avsian-Kretchmer et al., 2004; Xie et al., 2018; Pardo-Hernandez et al., 2020), and plant hormones play essential roles in mediating plant responses to ionic and osmotic stresses (Dai et al., 2018; Liu et al., 2022; Primo-Capella et al., 2022; Yacoubi et al., 2022), which possibly could help to improve tolerance to salt stress. However, the cellular pathways involved in the plant response to salt stress are still being explored. Plants usually react to stressful situations and improve their defensive ability by retaining and sustaining memories of the stress, allowing for stronger or faster responses to repeated instances of stress (Sharma et al., 2022). However, plant adaptation to environmental stress requires short and long term intercellular, inter-tissue, and inter-organ communication mediated by different signaling systems. Many studies of transcriptional changes (Peng et al., 2014; Tan et al., 2015; Robertson et al., 2017; Lee et al., 2021; Nivedita et al., 2021) have identified various plant tissue responses to salt stress, but an analysis of transcriptional changes triggered by a stress-induced vascular signal system in woody plants has not been done.

Dodder (*Cuscuta spp.*) is a vine plant with a parasitic stem without leaves and roots. It uses a unique organ, the haustorium (Yoshida et al., 2016), to attach to and penetrate the host’s stem tissue forming a connection between the xylem and phloem and the host’s vascular system (Shimizu and Aoki, 2019). The dodder parasitizes the plant host and steals nutrients and water from its vascular system, and exchanges a variety of molecules with it, including proteins, mRNAs, and small RNAs (Liu et al., 2020; Shen et al., 2020; Song et al., 2022). Research has revealed that systemic vascular signals can be delivered to another parasitized plant host through the dodder-bridged stem, which induces a response due to a biotic or abiotic stressor in the other host (Qin et al., 2019; Li et al., 2020; Zhang et al., 2021; Song et al., 2022). For example, salt treatment of one cucumber plant can prime the salt stress tolerance of another dodder-linked host and induce physiological and biochemical changes, including changes in the proline content, stomatal conductance, and the photosynthetic rate (Li et al., 2020). Signal transduction through the dodder can induce transcriptome and methylation changes in a host in a cucumber with a nitrogen deficiency, but not the response of the dodder with a nitrogen deficiency (Zhang et al., 2021). A biotic stress signal resulting from a pest attack, can also activate the dodder and cucumber defense responses. For instance, an aphid (*Myzus persicae*) feeding on the dodder can induce a systemic defense response in soybean, leading to increased host resistance to the insect (Song et al., 2022). The dodder stems often establish bridge links to multiple adjacent plants, establishing a plant community, i.e., a group of plants which consists of a single dodder and two or more host plants. Within these clusters, the dodder can likely transfer systemic molecular signals among the host plants. Further research has shown that many mRNAs, portable proteins, and microRNAs are transferred among the host plants, the dodder, and pests (Liu et al., 2020). Studies have also found that the dodder delivered mature proteins instead of mRNA from the host plant and still maintained the activity of mobile proteins. For instance, the FT protein synthesized by the host, not the dodder’s own, delivered into the dodder’s vascular system can interact with FD transcription factors to promote dodder self-flowering (Shen et al., 2020). The mobile system signal plays a critical role in triggering the response to biotic or abiotic stress and regulating growth and development. However, it is difficult to extract and analyze vascular system signals or inter-plants. A dodder-mediated signal analysis method provides an essential tool for this puzzle.

The research on dodder-mediated herbaceous plants is well developed, but similar research on woody plants with respect to salt stress is still lacking. Previous studies provide evidence for salinity-triggered transcriptomic profiling in citrus (Tan et al., 2015; Xie et al., 2017). However, no vascular system signal studies with citrus have been reported. Therefore, in this study, we analyzed the transcriptome profiles of salt-treated and dodder bridge-linked citrus plants. The transcriptional changes and reconfiguration in dodder-linked citrus leaves could reveal the abiotic-induced systemic signals of citrus plants, providing new insight and methods vis-à-vis dodder-mediated plant-plant interactions in woody plants.

## Materials and methods

### Plant materials preparation

**FIGURE 1:**
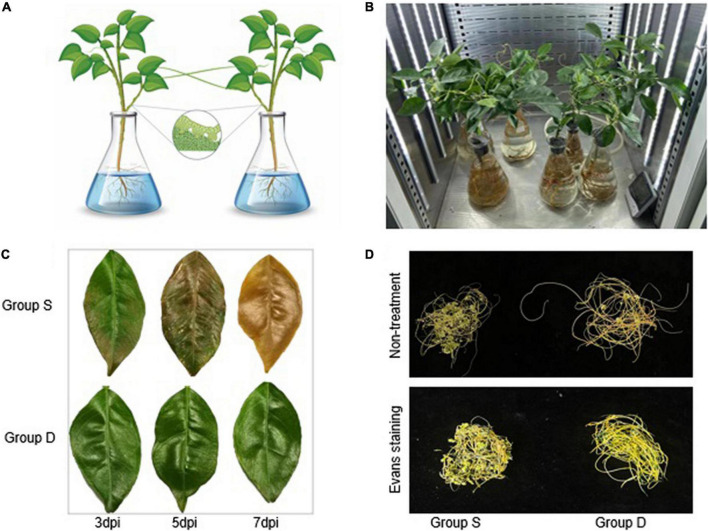
Experimental setup in this study. **(A)** Schematic of the experimental design for citrus-dodder-citrus community. **(B)** Group D, Group S, Mock, and the control group were set up in the growth chamber for phenotypes observation. The control group was mock-treated, while in group S, the citrus was treated with 500 mM NaCl. **(C)** The experimental groups citrus leaves for electrolyte leakage to monitor the cell viability during salinity treatment. **(D)** The dodder phenotypic observation in Groups S and D at 72 hpi.

The Hamlin sweet oranges (*Citrus sinensis* (L.) Osbeck) used in this study were kept in a greenhouse at the National Navel Orange Engineering Research Center, Gannan Normal University, Ganzhou, Jiangxi, China (with a 16-h light and 8-h dark photoperiod, a 25/18°C temperature cycle, at 50% humidity). Citrus plants (approximately 2 years old) were further cultured in modified Hoagland solution (MHS) (#NS10205, Coolaber, China). The dodders were germinated and used to parasitize periwinkle (*Catharanthus roseus* (L.) *G. Don*) to form stock. Periwinkle twined with dodder stocks were moved next to a citrus plant to parasitize the citrus stem. To understand the response and adaptation of the dodder to the stress signal *via* mobile signal mediation, a citrus community was constructed using hydroculture and dodder parasitism for subsequent transcriptional data analysis. The citrus was cultured in a Hoagland culture solution (#NS1011, Coolaber, China), and the adjacent plants were connected by a dodder to construct the citrus-dodder-citrus experimental sets (Figure 1A). Once infested with dodders, the citrus plants were transferred to a 500 mL conical flask containing MHS liquid medium. Citrus communities link-bridged with dodders were constructed by culturing two or more plants next to each other. For approximately 30 days, all community members solidly infested by dodders were used for salinity treatment experiments. Then one of the citrus community plants was infiltrated with 500 mM NaCl in Hoagland medium for phenotype observation and continuous sample collection (Figure 1B). Subsequently, two-time points [12 and 24 h post immersion in the salt solution (hpi)] were chosen for transcriptional analysis. The experimental groups were designed as follows: the salinity-treated groups of sweet orange seedlings, as group S, and the dodder bridge-linked experimental group of sweet oranges, as group D, no salinity-treated groups of sweet orange seedlings, as mock.

### RNA extraction, reverse transcription, and illumina sequencing

Leaf samples were collected, frozen in liquid nitrogen, and stored at –80°C until RNA extraction was carried out. RNA extraction was performed on all plant tissues using an RNA isolation kit (#DR0409050, Easy-Do, China). The absorbance ratio was determined *via* a Nanodrop spectrophotometer (NanoDrop Technologies, Wilmington, United States) to assess the RNA quality and concentration. RNA samples (OD260/280 > 2.0 and OD260/230 > 2.0) were used for further analysis. The first-strand cDNA was synthesized from RNA samples using an RNA reverse transcription kit (#RR047A, Takara, Japan) according to the manufacturer’s instructions. The resultant cDNA was purified and enriched by PCR to produce the final cDNA libraries. The libraries were sequenced on an Illumina Hiseq 2000 platform, and 100 bp paired-end reads were generated for transcriptome sequencing.

### Analysis pipeline

Clean data and count numbers of the RNA sequencing libraries were assessed and summarized using the transcriptional analysis website Dr. Tom.^1^ The statistical number of genes in different TPM (Transcripts Per Kilobase of exon model per Million mapped reads) ranges for each sample was assessed for three ranges (TPM ≤ 1, TPM 1 –10, TPM ≥ 10) (Supplementary Figure 1). After alignment, the mapping rate statistics and the distribution of reads on the reference sequence were used to determine whether the alignment result passed a second QC. A gene quantification analysis and other analyses based on gene expression (i.e., principal component, correlation, differential gene screening) were then carried out, and a significant enrichment analysis of gene ontology (GO) functions for the DEGs among the screened samples, a significance enrichment pathway analysis, clustering, protein interaction networks, and transcription factors, and a more in-depth mining analysis were performed.

### Categorization of differentially expressed genes

The RNA-seq data was loaded into the software with a filter of FDR < 0.05, The *Q*-value was obtained by correction of the *P*-value (the function of *Q*-value ≤ 0.05 was regarded as a significant enrichment). For the MapMan analysis, all gene IDs were transferred into the corresponding citrus microarray library. The pathways were visualized in the context of built-in Citrus AFFY mapping, and a Wilcoxon rank-sum test implemented in MapMan was used to extract the item number in each BIN and return a BIN whose gene members showed significantly different gene expression. To analyze GO term enrichment of differentially expressed genes, SEA was performed online through the agriGO program, a GO analysis tool kit for the agricultural community.^2^ Briefly, the differentially expressed gene probe identification numbers were first uploaded into agriGO; then, the Citrus Affymetrix Genome Array was selected as the reference. Statistical *P*-values were calculated with the Fisher multiple-test adjustment using the Yekutieli (FDR under dependency) method. Venn diagrams were generated using an online program, Draw Venn Diagram.^3^

### Quantitative reverse transcription-polymerase china reaction analyses

All primers used for quantitative reverse transcription-polymerase china reaction (qRT-PCR) in various experiments were designed on the website^4^ using the following parameters: melting temperatures between 60 and 64°C, oligo lengths of 18–30 bp, a GC contents of 40%–60% and an amplicon sizes between 70 and 150 bp. An ABI QuantStudio5 Real-time PCR system (#A28139, Thermofisher, United States) was used to perform qRT-PCR using a 2 × SYBR Green qPCR Mix (#SL2690, Coolaber, Beijing, China). Each reaction was run in a 10-μL reaction system containing 2-μL of template cDNA, 5-μL of SYBR Green qPCR mix, and a final primer concentration of 300 nm. All reactions were performed under the following conditions: 30 s at 95°C and 40 cycles of 5 s each at 95°C and 30 s at 60°C in 96-well optical reaction plates (#4346907, applied biosystems, United States). A melting curve was generated from 60 to 95°C at the end of the reaction. The gene expression levels in all samples were determined by the number of cycles (Ct) required for the amplification-related fluorescence to reach a specific threshold detection level. The raw Ct value was analyzed using QuantStudio™ design and analysis software v1.4.1. The 2^–ΔΔ*Ct*^ method was applied for relative quantification. Each experiment was repeated twice, and contained three replicates. The endogenous housekeeping gene was GAPDH (glyceraldehyde 3-phosphate dehydrogenase) (NCBI: LOC102614495) and the gene-specific primer sequences and information are listed (Supplementary Table 6).

### Electrolyte leakage determination

Five citrus leaves were collected from each experimental plants. All leaves were wiped, rinsed with ddH_2_O three times, and then soaked in 30 mL of ddH_2_O. After immersion in a 37°C incubator with shaken at 200 rpm for 24 h, the electrolyte leakage value (L_t_) of leaves was measured using a conductometer (#DDSJ-308F, INESA, Shanghai, China). The total electrical conductivity (L_0_) was obtained after the same samples were heated in a water bath at 90°C for 2 h. The relative electrolyte leakage (L) was calculated using the formula:

$$L=L_{t}/L_{0}\times 100\%.$$

### Evans blue assay

Five citrus leaves were soaked into ddH_2_O and then immersed in a 37°C incubator shaken at 200 rpm for 12 h. All leaves were immersed in a 0.5% (w/v) Evans blue solution (#SL7200, Coolaber, Beijing, China) for 24 h at room temperature. Each leaf was rinsed extensively with ddH_2_O and photographed. Then the leaves were immersed in an ethanol series (95–25%) with an 8 h interval between concentrations, and photographed again.

### Statistical data analysis

Statistical analysis was performed with the Prism 9 software (Graphpad, United States). All numerical data were expressed as mean ± standard deviation (SD) unless specified. The *P*-values and statistical analysis methods are indicated. One/two-way ANOVA analyses were processed with corresponding subsequent multiple comparison tests to meet the data analysis requirement with the Tukey test (95% confidence interval) using GraphPad Prism 9 (**p ≤ 0.05, ***p ≤ 0.001, ****p ≤ 0.0001, ns *P* > 0.05).

## Result

### Experimental design and phenotypic observation

A citrus community for subsequent transcriptional data analysis was constructed using hydroculture and dodder parasitism. One of the citrus community plants was then infiltrated in a Hoagland medium with 500 mM NaCl for phenotypic observation and continual sample collection (Figure 1B). The phenotypic observation results showed that the haustoria of the dodder easily separated from the stems of the group S at 48 hpi. The dodder parasitized on the stem of group S evidenced a weakly salt-stressed phenotype at 72 hpi, while the dodder linked to the stem of group D grew normally (Figure 1D). The leaves of the orange plant in the group S showed fallen, wilting, greenness, or necrotic salt stress-related phenotypes at 7 days post-treatment, while the leaves of the group D did not show any abnormal phenotypes. The photosynthetic intensity of the leaves of the experimental groups was measured every 2 h. To monitor the cell viability during the salt treatment, we determined the electrolyte leakage to the leaves and dodders in the experimental groups at 7 days post-treatment. The results showed that the leaves from group S were heavy, but those from group D were barely damaged (Figure 1C). The ion leakage from the leaves of group S ranged from 67 to 72.13%, approximately 70.47% higher than in the leaves of group D. Interestingly, the dodder parasite associated with group S showed apparent growth retardation, evidencing a weak salt-stress phenotype compared with the dodder parasite associated with group D. However, the dodder parasite associated with group S showed similar cell viability to the dodder parasite associated with group D during the leakage detection experiment. The Evans blue staining assay revealed that the linked dodder in both experimental groups retained complete cell vitality during salt stress.

### RNA-seq quality statistics

In order to analyze the transcriptional changes in sweet orange leaves in response to salt stress, the leaves of group S and the dodder were collected for transcriptome analysis at 0 h, 12 h, and 24 h post salt treatment.

In this study, The NCBI Reference Genome Version Csi_valencia_1.0^5^ was employed. Eighteen samples were sequenced using the DNBSEQ platform (sequencing length PE150), with an average yield of 6.74 Gb data per sample (Supplementary Table 1). We used HISAT to align the clean reads to the reference genome. We aligned the clean reads to the reference genes using Bowtie2 to obtain the alignment result (Supplementary Table 2). The average alignment ratio of the sample comparison genome was 88.09%, which was comparative. The average alignment of the gene sets was 77.47%, and 22,465 genes were detected. The coverage of the transcripts of each sample in this project based on the alignment results is shown (Supplementary Figure 2). A boxplot of an expression quantity analysis combined with a sequencing saturation analysis depicted the distribution of gene expression levels in each sample. The dispersal degree of the data distribution was stable and equal (Supplementary Figure 2), revealing that sequencing data met the experimental requirements. Additionally, a density map analysis showed a parallel trend of gene abundance in the samples as the expression level changed, reflecting that the interval of the gene expression concentration among samples were similar (Supplementary Figure 3).

A principal component analysis (PCA) is a multivariate statistical analysis method that reduces multiple variables to a small number of independent variables (principal components) by reducing the dimensionality while retaining as much of the original information as possible. Our quality analysis revealed that the filtering and mapping read of the RNA-seq data represent an acceptable sequencing standard among the different samples, allowing a PCA and Heatmap analysis using Pearson correlation coefficients to be applied. The PCA facilitated the identification of outlier samples and the discrimination of sample clusters with high similarity, revealed the variance among each group and indicated that group D was similar to group C at 12hpi (Supplementary Figure 4). The Pearson correlation coefficients for all gene expressions between each samples pair were subsequently calculated, and these coefficients were plotted as a heatmap (Supplementary Figure 5). A Heatmap of the expression profiles of the DEGs (Filtered with | log2FC| ≥ 1.58, *Q*-value ≤ 0.05) was used to validate RNA-Seq data obtained from both experimental groups (424 genes were defined after the *t*-test) (Supplementary Figure 6). Results showed that the correlation heatmap results were consistent with the PCA and showed better homogeneity within experimental groups, which indicated that the read number and quality were sufficient for further analysis.

### Dynamic transcriptome response of citrus and dodder to salt stress

**FIGURE 2:**
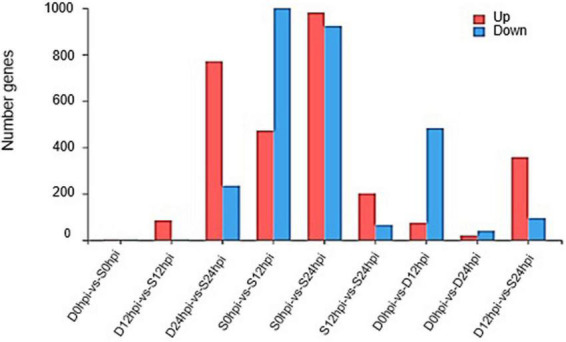
Changes in gene expression profile. The DEGs numbers of up-regulated and down-regulated genes among groups S and D at different time points are summarized.

Quantification of transcript abundance using standardized TPM helped us compare mRNA levels among samples. The genes with a cutoff | log2FC | ≥ 1.58 and *Q*-value ≤ 0.05 were defined as DEGs. A total of 1,472 (472 up-regulated, 1,000 down-regulated) and 1,904 (981 up-regulated, 923 down-regulated) DEGs at 12 hpi and 24 hpi were identified in the salt group, and 557 (74 up-regulated, 483 down-regulated) and 59 (19 up-regulated, 40 downregulated) genes were identified in group D at 12 hpi and 24 hpi. Groups S and D shared 424 DEGs at 12 hpi, accounting for 76.12% of the total DEGs in group D, indicating a high correlation and cooperativity in a weighted correlation network analysis (Supplementary Figure 7). After 24 h, group S showed more obvious expression patterns related to salt stress than at other time points, and more obvious differences in the regulation of gene expression (Figure 2). Interestingly, the gene expression of the Group D showed an obvious adaptation to salt stress, and the regulation of gene expression was consistent with the initial gene expression pattern.

**FIGURE 3:**
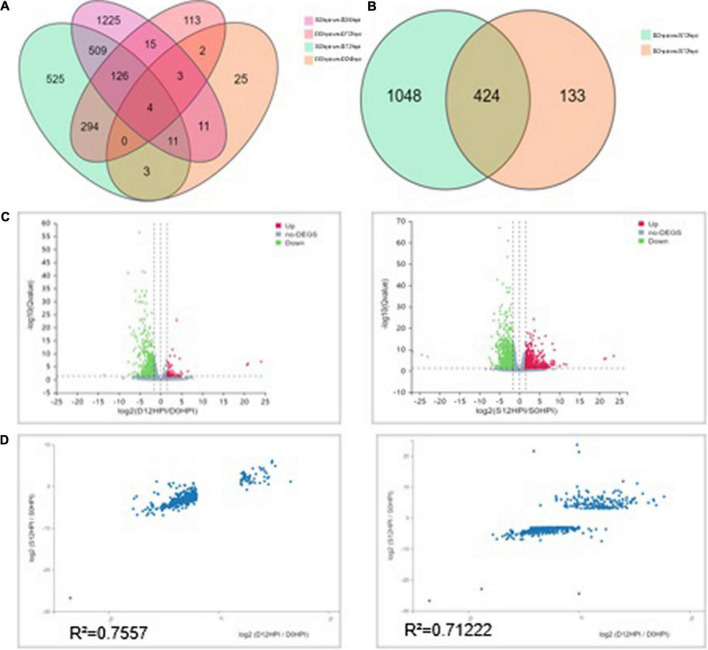
Differentially expressed genes (DEGs) analysis among groups at different time points. **(A)** Venn diagram showing the relationship between the differentially expressed genes at 12 and 24 hpi, respectively. The values in each circle represent the number of genes. The cut-off value of log2 fold changes for up-regulated and down-regulated gene/protein was ±1.58. **(B)** The Venn diagram distribution of DEGs in groups S and D at 12hpi. **(C)** Volcano plot of DEGs signals in groups S (left) and D (right). The scatter in the figure represents each gene; green genes are down-regulated, red genes are up-regulated, and gray genes are not differentially expressed. The vertical axis indicates the *P*-value (−log 10 value), | log2FC| ≥ 1.58 were applied for the cut-off value. **(D)** Correlation between the DEGs (| log2FC| ≥ 1.58) of groups S (left) and D (right) at 12hpi. The value represents 557 DEGs in group D (Left) and 1,472 in group S (Right). The regression line and Pearson’s correlation coefficient are shown.

Despite the overlapping genes, a dramatic difference was observed in the transcriptional profiles at 12 hpi and 24 hpi. At 12 hpi, 424 DEGs were found in the group S and D transcriptomes (Figure 3B). However, at 24 hpi, the number of DEGs in the salt stress group and the Group D decreased to 29. There were only 59 DEGs in Group D at 24hpi, while there were 1,904 DEGs in group S. The overlapping differentially expressed genes at the two-time points were compared using a Venn diagram analysis (Figure 3A). Four genes were found to be regulated at all three-time points: the two down-regulated genes were the small heat shock protein (LOC102608585), and a zinc-transporting ATPase HMA3(LOC102625788), and the two up-regulated genes were the late embryogenesis abundant protein(LOC102616716) and an amino acid transporter AVT1C(LOC102623467), respectively (Figure 3A). The results showed no similarity of the transcriptional changes between the two groups after 24 h, but the gene expression changes were evident in the leaves of group S. In contrast, the gene expression of group D was consistent with that of the 0 hpi group, indicating that the leaves of group D had adapted to the early salt stress signal. A combination of a volcano plot and a correlation analysis at 12 hpi indicated the most of the DEGs identified in the group S and group D at 12 hpi showed a consistent, similar gene expression trend (Figures 3B,C,D), indicating that the system signal triggered by salt in the root of group D was transmitted through the dodder and citrus vascular tissue and induced transcriptional changes in the leaves of group D. Therefore, 12 hpi is the critical time for analyzing the stress-signal transduction transcriptional regulation in the following contents.

### Signaling pathways are involved in salt stress

To compare the DEGs at 12 hpi and 24 hpi, we analyzed the citrus genes whose mRNA levels were altered in response to salt stress in group S and group D. We further assigned the DEGs to functional categories according to their similarity to genes with known functions in plants using two separate software packages: the MapMan software and the web-based singular enrichment analysis (SEA) tool in the AgriGO software (see text footnote 2). According to the AgriGO-SEA interpretation, the enrichment or over-representation of a category was assessed with a significantly (FDR < 0.05) higher percentage in all of the DGEs than in the overall percentage in the total expressed sequence tag. At 12 hpi, the downregulated genes in group S were enriched for photosynthesis, response to stimulus, cellular nitrogen compound metabolic process, and pigment metabolic process. Interestingly, compared to group S, the DEGs of group D at 12hpi showed similar enrichment for photosynthesis and response to stimulus. At 12 hpi, the singular enrichment analysis of significant terms of DEGs in group S revealed that the categories of “response to the stimulus” (FDR:0.00042, GO:0050896), “response to biotic stimulus” (FDR:0.00027, GO:0009628), “cellular response to stimulus” (FDR:0.041, GO:0051716) and “response to stress” (FDR:0.023, GO:0006950) were the significant terms. In group D, “response to stimulus” (FDR:0.029, GO:0050896) was the only significant term under the analysis (Supplementary Figure 8). The singular enrichment analysis of cellular components in groups S and D was consistently localized in the photosynthetic membrane, photosystem I, Photosystem II, plastid, and chloroplasts (Supplementary Figure 9).

We then used AgriGO-SEA to interpret this time-based comparison, which illustrated that most of the differentially expressed gene-enriched categories at 12 hpi were consistent with a comparison of groups S and D with respect to biological process cellular components. The AgriGo analysis results indicated that significantly down-regulated gene expression was enriched for photosystem I and photosystem I thylakoids, photorespiration, and the Calvin cycle in the chloroplasts of group S. Compared with group S, only photosystem II was affected in group D during salt stress (Supplementary Figure 10). A Wilcoxon rank-sum test of the cellular response overview revealed that biotic stress, abiotic stress, and development were highly enriched in DEGs of both groups at 12 hpi.

### Gene ontology term enrichment with respect to the salt stress-response

**FIGURE 4:**
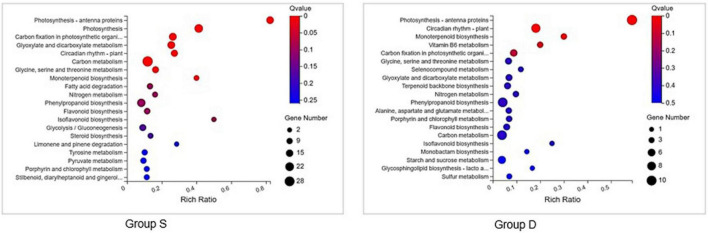
KEGG pathway enrichment scatters plot of experimental groups at 12hpi. Top 20 enriched pathways of KEGG metabolism pathway categories of DEGs in groups S (left) and D (right). The vertical axis represents the pathname, and the horizontal axis represents the path factor corresponding to the Rich factor. The color of the point represents the size of the *q*-value. The number of differential genes included in each pathway is expressed by the point’s size. (The smaller the *Q*-value, the closer the color is to the red color).

**FIGURE 5:**
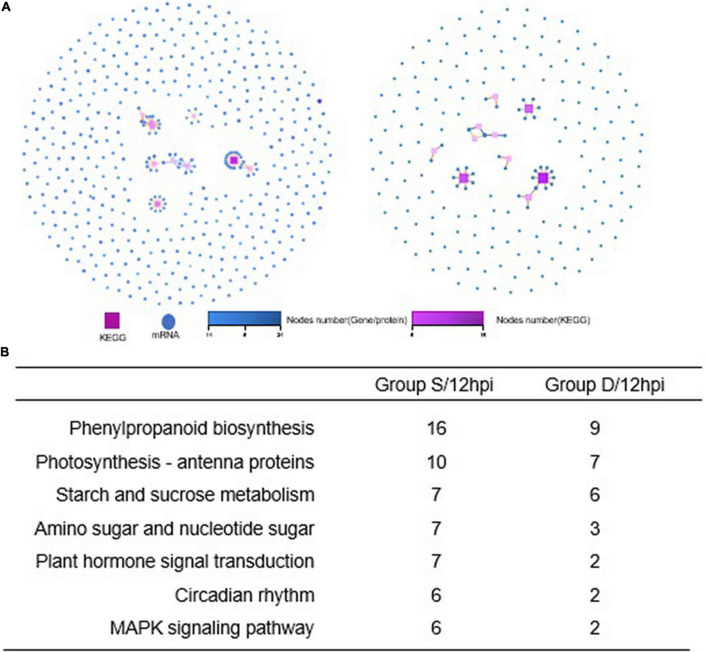
KEGG pathway relationship network analysis and same nodes comparison between experimental groups at 12hpi. **(A)** The KEGG pathways of the selected genes are ranked by the number of genes on the pathway, and the top 10 pathways with the most significant number of genes were displayed in Group S (left) and Group D (right), respectively. Different shapes represent different content (nodes); the box represents KEGG Pathway, and the circle represents mRNA. Both color and size indicate the number of genes or transcripts connected to the node. The darker the color, the size of the box, the line with a different color represents the different path classification, and red represents cellular processes. **(B)** The nodes gene between groups S and D at 12hpi in KEGG pathway relationship networks.

A GO analysis was performed on the DEGs at 12 hpi between group S and group D, including “main metabolic process,” “nitrogen compound metabolism process,” and “cellular macromolecular biosynthesis process.” The second GO class enrichment statistics are for the 424 DEGs in group D and group S at 12 hpi (Filtered with | log2FC| ≥ 1.58, *Q*-value ≤ 0.05). The results showed that KEGG pathways, such as phenobarbitone biosynthesis, photosynthesis, carbon fixation in the photosynthetic organization, circadian rhythm, and carbon metabolism, were enriched, suggesting that these pathways may be involved in the signal regulation of early system responses in plants (Figure 4). A KEGG pathway relationship network analysis showed the main significantly up-regulated categories were related to phenylpropanoid biosynthesis, photosynthesis-antenna protein, starch and sucrose metabolism, amino nucleotide sugar, plant hormone signal transduction, circadian rhythm, and the MAPK signaling pathway in both group S and group D (Figure 5).

According to the GO annotation classification, the phyper function in the R software was used to perform an enrichment analysis and calculate the *P*-value. When comparing the up-and downregulated genes in both group S and group D at different time points, we found that the biological process categories of photosynthesis, light-harvesting, protein-chromophore linkage, and response to light stimulus were enriched for all of the down-regulated genes in both group D and group S at 12 hpi, and no significant enrichment categories were found for all up-regulated genes in both groups (Supplementary Table 3). We found that the molecular function categories of chlorophyll-binding, channel activity, water channel activity, and oxidoreductase activity were enriched in both groups. No significant enrichment in up-regulated genes was found in either group (Supplementary Figure 11). Consistent with the number of identified DEGs, a Pearson correlation analysis based on the TPM values of all DEGs also showed significant differences in expression between the transcriptomes of the different leaf samples at 24 hpi, but no significant enrichment of biological processes or pathways. However, the DEGs in group S and group D were enriched in several processes at 12 hpi, including “metabolic process,” “cell process,” and “stress response.” The cellular component categories enriched in all down-regulated genes of group D are plastoglobule, plastid, photosystem, thylakoid, chloroplast, chloroplast thylakoid membrane, photosystem II, an integral membrane component, and chloroplast envelope, which is consistent with the down-regulated genes enriched in group S.

### The set of key driver salt stress-responsive genes

The key driver gene analysis (KDA) was employed in this study to predict the node genes through the PPI relationship, which can help in understanding the regulatory relationship with the altered gene set and can regulate the main gene/protein at 12 hpi (Filtered with | log2FC| ≥ 1.58, *Q*-value ≤ 0.05). Filtering the results showed that 10 key driver genes and 147 linked genes were predicted in group S, and 10 key driver genes and 171 linked genes in group D (Supplementary Figure 12). The most node genes were highlighted in chloroplasts and regulated the binding/catalytic activity functions on chlorophyll-binding (GO:0016168), phosphatase activity (GO:0016791), phosphoglycolate phosphatase activity (GO:0008967), fructose 1,6-bisphosphate 1-phosphatase activity (GO:0042132), magnesium-protoporphyrin IX monomethyl ester (oxidative) cyclase activity (GO:0048529) and ribulose-bisphosphate carboxylase activity (GO:0016984). The KEGG network and KDA analysis indicated that node genes are consistent with the biological photosynthesis process, which hinted that critical biological processes are regulated by the vascular signal triggered by salt treatment in *C. sinensis.*

### Stress-related secondary metabolism pathway induced by the dodder-delivered signals

**FIGURE 6:**
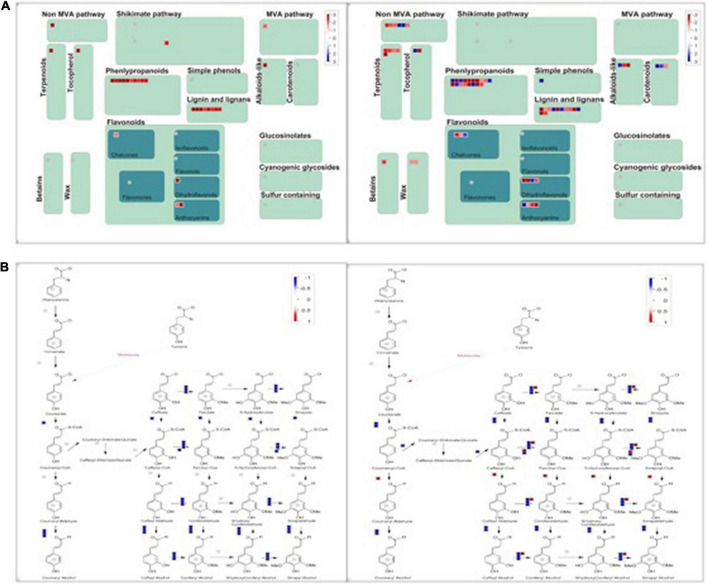
MapMan-based visualization of the transcripts involved in “secondary metabolism” in experimental groups (a). **(A)** The secondary metabolism pathway compares groups S (left) and D (right) at 12hpi. Functional subBINs shown in red or blue indicate up/down-regulation, respectively. **(B)** All DEGs involved in the pathway of phenylpropanoids in both groups S (left) and D (right) at 12 hpi. **(B)** The DEGs are involved in the Phenylpropanoids synthesis Pathway.

Phenylpropanoids have been reported to play an essential role in plant adaptation against salt stress. The Phenylpropanoid pathway transforms phenolics, flavonoids, anthocyanins, and lignin, and it can help the plant adapt to abiotic stress. The chemical structures of phenylpropanoids can facilitate the degradation of reactive oxygen species by reducing free radicals. A MapMan Wilcoxon Rank Sum analysis found that the significantly regulated genes were involved in stress, the photosystem, secondary metabolism, hormone metabolism, TCA, and fermentation in group D. The significantly regulated categories included the photosystem, stress, protein, tetrapyrrole synthesis, and major CHO metabolism in group S. We visually assessed the differentially expressed genes in a MapMan pathway, which revealed that the categories phenylpropanoids, lignin, and lignans contained remarkably more regulated genes than other secondary metabolism pathways in both groups (Figure 6). Therefore, the activation of the phenylpropanoid genes is concomitant with the detection of stress in plants. In both groups, the phenylpropanoids synthesis process was significantly and comprehensively changed during salt stress at 12 hpi.

### Quantitative reverse transcription-polymerase reaction verification

**FIGURE 7:**
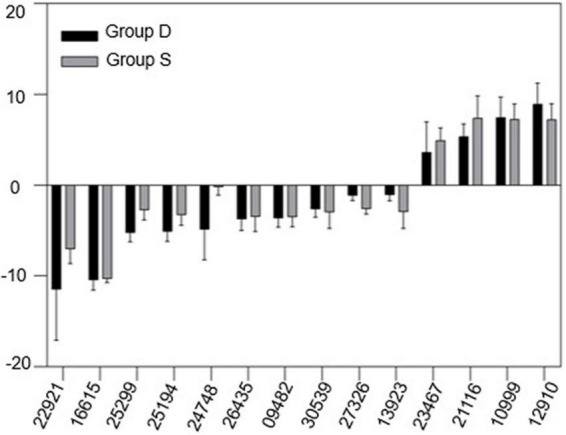
Gene expression change between group D and S. mRNA samples were extracted from the leaves of both experimental groups at 12hpi. The housekeeping gene GAPDH was used as an endogenous control. Each value represents three replicates mean ± standard deviation (SD).

Our previous analysis suggested that vascular system signals can be triggered from the rootS of group S, then transmitted *via* the dodder stem and delivered into the leaves of group D in 12 h. The transcriptional level in the leaves of group D showed a consistent change with group S at 12 hpi but became different at 24 hpi. Therefore, qRT-PCR was used to validate the RNA-seq data and our hypothesis. A total of 14 overlapping DEGs between two experimental groups at 12 hpi, overlaying DEGs were selected from the RNA-seq data, based on the KDA and the pathway network analysis results (Supplementary Table 4). The qRT-PCR results showed a similar trend in the gene expression pattern and abundance with the RNA-Seq data (Figure 7), which further confirmed the reliability of the RNA-seq data and the above analysis results.

## Discussion

A dodder connects to the vascular tissue of a host plant through the haustoria, which not only absorbs nutrients but also transmits systemic signaling molecules between separate hosts. The dodder has long been used in citrus research and is a critical vector for studing the spread and expansion of the most important bacterial disease of citrus, the citrus huanglongbing (HLB) pathogen (Zhou et al., 2007; Duan et al., 2008). However, there is no precedent for the application of a dodder in signal transmission between citrus plants. Using a dodder as a signal transduction bridge between herbacula hosts, has recently become an essential tool to study signal transduction in herbacula with respect to abiotic/biotic stress. In this study, the dodder was used to study the transcriptional signaling system of plants with respect to salt stress on a separate host, the woody plant- *C. sinensis*, which not only indicated that dodder could transmit the salt stress signals over a long distance but also showed that the signal-regulated transcriptional changes were induced in dodder bridge-linked hosts. Our results suggest that a regulatory role for signals in citrus in response to salt stress and provide a reliable experimental system for woody plants to respond to various stress responses. Previous research (Li et al., 2020) revealed that the Na^2+^ ion was not delivered through the vascular system of the dodder or the host plant. Therefore, it was the salt particles themselves that caused the salt stress phenotype in the dodder, rather than systemic vascular signals generated after salt stress in sweet orange. The stress signal can be transferred to the vascular tissue of orange through the stem of the dodder, subsequently inducing a transcriptional response to salt stress signal.

A KEGG enrichment analysis revealed that photosynthesis, emergency response, and rhythm genes were significantly enriched. In addition, using a MapMan analysis, we found that the phenylpropanoid, lignin, and lignan pathways were significantly regulated in both groups. Phenylpropanoid compounds play an essential role in plant adaptation to different environmental conditions. Abiotic stress usually stimulates the phenylpropanoid pathway as evidenced by increases in phenols, flavonoids, anthocyanins, and lignin. These compounds protect plants from diseases or pests. The phenylpropanoid chemical structure can reduce reactive oxygen species by reducing free radicals. Therefore, the activation of the phenylpropanoid gene concomitant with stress induction in plants. Understanding the regulation of the phenylpropanoid pathway might be used to improve cultivation of crops, even under limiting stress conditions. Light, temperature, drought, and salt are stressors that activate the amphetamine gene. In recent years, microRNAs have been shown to regulate the phenylpropanoid genes, and synthetic microRNAs can also be used to regulate the expression of the phenylpropanoid genes. In addition, lignin biosynthesis genes play an essential role in plant adaptation to extreme salt stress. A metabolic analysis of a suspension cell culture of Arabidopsis thaliana root adapted to high salt stress showed that the lignin content increased and the cell wall became thickened. The regulation of the secondary metabolic pathway presents the appearance of salt tolerance and adaption characteristics in all experimental groups, which revealed an early salt stress response triggered by vascular system signal in citrus and provided new insight for improving plant stress resistance.

A dodder may transmit various stress-induced signals between host plants, changing the interaction or adaptation mode between the host plants and the environment. This study suggests that a dodder can form a “telephone wire” to convey stress response between woody citrus plants under saline conditions. This study is significant to our understanding of the periodic signals of stress and provides a theoretical basis for an agricultural understanding of the early response regulation of *C. sinensis* to salt stress. Additionally, this study provides a theoretical timepoint of the system’s signal generation and transmission characteristics in response to stress in the group S plants. Furthermore, the transcriptional change triggered by dodder-mediated mobile signal has been proposed, which helped understand the vascular stress-induced vascular signals at an early stage in woody plant. The other potential application of this study lies in the related research of citrus bacterial diseases (e.g., citrus bacterial canker disease and HLB). HLB is a devastating citrus disease caused by the phloem-colonizing bacterium “*Candidatus* Liberibacter asiaticus” (CLas). As a phloem-restricted bacteria, the citrus HLB pathogens can not be cultured *in vitro*, and its pathogenic mechanism is not clearly understood. A dodder can transmit the HLB pathogens from an infected to a healthy tree, which suggests that the dodder has potential for significant application in the study of pathogenic mechanism of citrus HLB. Recently, HLB has been considered as a pathogen-triggered immune disease, suggesting a new research direction for HLB studies, such as vascular signal transduction in and related triggering of an immune response (Ma et al., 2022). The dodder has not been used as a tool for signal transduction in woody plants because an effective model for its use has not been constructed. This study established a salt-mediated vascular signal transport system among a citrus-dodder-citrus community and a corresponding transcriptome change, which will provide an experimental basis for subsequent studies of citrus bacterial diseases.

## Data availability statement

The original contributions presented in this study are publicly available. This data can be found here: NCBI, PRJNA839513.

## Author contributions

SD and M-LB: conceptualization. SD, M-LB, and ZX: writing – original draft preparation. H-JM and W-SD: writing – review and editing. SD: supervision and project administration. ZX: transcriptome analysis. X-YL, PL, M-LB, and SD: experiment. H-KQ: plants maintenance. All authors have read and agreed to the published version of the manuscript.

## Footnotes

1
https://biosys.bgi.com/

2
http://systemsbiology.cau.edu.cn/agriGOv2/index.php

3
https://www.vandepeerlab.org/

4
https://www.idtdna.com/Primerquest/Home/Index

5
https://www.ncbi.nlm.nih.gov/assembly/GCF_000317415.1

## References

[B1] Arif Y. Singh P. Siddiqui H. Bajguz A. Hayat S. (2020). Salinity induced physiological and biochemical changes in plants: an omic approach towards salt stress tolerance. *Plant Physiol. Biochem.* 156 64–77. 10.1016/j.plaphy.2020.08.042 32906023

[B2] Avsian-Kretchmer O. Gueta-Dahan Y. Lev-Yadun S. Gollop R. Ben-Hayyim G. (2004). The salt-stress signal transduction pathway that activates the gpx1 promoter is mediated by intracellular H2O2, different from the pathway induced by extracellular H2O2. *Plant Physiol.* 135 1685–96. 10.1104/pp.104.041921 15247370 PMC519082

[B3] Chaves M. Flexas J. Pinheiro C. (2009). Photosynthesis under drought and salt stress: regulation mechanisms from whole plant to cell. *Ann. Bot.* 103 551–60. 10.1093/aob/mcn125 18662937 PMC2707345

[B4] Dai W. Wang M. Gong X. Liu J. (2018). The transcription factor FcWRKY40 of Fortunella crassifolia functions positively in salt tolerance through modulation of ion homeostasis and proline biosynthesis by directly regulating SOS2 and P5CS1 homologs. *New Phytol.* 219 972–89. 10.1111/nph.15240 29851105

[B5] Duan Y. P. Gottwald T. Zhou L. J. Gabriel D. W. (2008). First Report of dodder transmission of ‘*Candidatus* Liberibacter asiaticus’ to tomato (*Lycopersicon esculentum*). *Plant Dis.* 92:831. 10.1094/PDIS-92-5-0831C 30769608

[B6] Golldack D. Li C. Mohan H. Probst N. (2014). Tolerance to drought and salt stress in plants: unraveling the signaling networks. *Front. Plant. Sci.* 5:151. 10.3389/fpls.2014.00151 24795738 PMC4001066

[B7] Lee C. Chung C. T. Hong W. J. Lee Y. S. Lee J. H. Koh H. J. (2021). Transcriptional changes in the developing rice seeds under salt stress suggest targets for manipulating seed quality. *Front. Plant Sci.* 12:748273. 10.3389/fpls.2021.748273 34819939 PMC8606889

[B8] Li S. Zhang J. Liu H. Liu N. Shen G. Zhuang H. (2020). Dodder-transmitted mobile signals prime host plants for enhanced salt tolerance. *J. Exp. Bot.* 71 1171–84. 10.1093/jxb/erz481 31665509 PMC6977188

[B9] Liu N. Shen G. Xu Y. Liu H. Zhang J. Li S. (2020). Extensive inter-plant protein transfer between *cuscuta* parasites and their host plants. *Mol. Plant.* 13 573–85. 10.1016/j.molp.2019.12.002 31812691

[B10] Liu W. C. Song R. F. Zheng S. Q. Li T. Zhang B. L. Gao X. (2022). Coordination of plant growth and abiotic stress responses by Tryptophan Synthase beta Subunit1 through modulating tryptophan and ABA homeostasis in Arabidopsis. *Mol. Plant* 2022:9. 10.1016/j.molp.2022.04.009 35488429

[B11] Ma W. Pang Z. Huang X. Xu J. Pandey S. S. Li J. (2022). Citrus Huanglongbing is a pathogen-triggered immune disease that can be mitigated with antioxidants and gibberellin. *Nat. Commun.* 13:529. 10.1038/s41467-022-28189-9 35082290 PMC8791970

[B12] Mahajan S. Tuteja N. (2005). Cold, salinity and drought stresses: an overview. *Arch. Biochem. Biophys.* 444 139–58. 10.1016/j.abb.2005.10.018 16309626

[B13] Nivedita R. A. Ramchiary N. Abdin M. Z. (2021). A high-throughput RNA-Seq approach to elucidate the transcriptional response of Piriformospora indica to high salt stress. *Sci. Rep.* 11:4129. 10.1038/s41598-021-82136-0 33602957 PMC7893156

[B14] Pandey P. Irulappan V. Bagavathiannan M. V. Senthil-Kumar M. (2017). Impact of combined abiotic and biotic stresses on plant growth and avenues for crop improvement by exploiting physio-morphological traits. *Front. Plant. Sci.* 8:537. 10.3389/fpls.2017.00537 28458674 PMC5394115

[B15] Pardo-Hernandez M. Lopez-Delacalle M. Rivero R. M. (2020). ROS and NO regulation by melatonin under abiotic stress in plants. *Antioxidants* 9:9111078. 10.3390/antiox9111078 33153156 PMC7693017

[B16] Peng Z. He S. Gong W. Sun J. Pan Z. Xu F. (2014). Comprehensive analysis of differentially expressed genes and transcriptional regulation induced by salt stress in two contrasting cotton genotypes. *BMC Genom.* 15:760. 10.1186/1471-2164-15-760 25189468 PMC4169805

[B17] Primo-Capella A. Forner-Giner M. A. Martinez-Cuenca M. R. Terol J. (2022). Comparative transcriptomic analyses of citrus cold-resistant vs. sensitive rootstocks might suggest a relevant role of ABA signaling in triggering cold scion adaption. *BMC Plant Biol.* 22:209. 10.1186/s12870-022-03578-w 35448939 PMC9027863

[B18] Qin Y. Zhang J. Hettenhausen C. Liu H. Li S. Shen G. (2019). The host jasmonic acid pathway regulates the transcriptomic changes of dodder and host plant under the scenario of caterpillar feeding on dodder. *BMC Plant Biol.* 19:540. 10.1186/s12870-019-2161-8 31801469 PMC6894313

[B19] Robertson L. S. Galbraith H. S. Iwanowicz D. Blakeslee C. J. Cornman R. S. (2017). RNA sequencing analysis of transcriptional change in the freshwater mussel Elliptio complanata after environmentally relevant sodium chloride exposure. *Environ. Toxicol. Chem.* 36 2352–66. 10.1002/etc.3774 28224655

[B20] Sharma M. Kumar P. Verma V. Sharma R. Bhargava B. Irfan M. (2022). Understanding plant stress memory response for abiotic stress resilience: molecular insights and prospects. *Plant Physiol. Biochem.* 179 10–24. 10.1016/j.plaphy.2022.03.004 35305363

[B21] Shen G. Liu N. Zhang J. Wu J. Q. (2020). Cuscuta australis (dodder) parasite eavesdrops on the host plants’ FT signals to flower. *Proc. Natl. Acad. Sci. U S A.* 117 23125–30. 10.1073/pnas.2009445117 32868415 PMC7502711

[B22] Shimizu K. Aoki K. (2019). Development of parasitic organs of a stem holoparasitic plant in genus *cuscuta*. *Front. Plant Sci.* 10:1435. 10.3389/fpls.2019.01435 31781146 PMC6861301

[B23] Song J. Bian J. Xue N. Xu Y. Wu J. Q. (2022). Inter-species mRNA transfer among green peach aphids, dodder parasites, and cucumber host plants. *Plant Divers.* 44 1–10. 10.1016/j.pld.2021.03.004 35281124 PMC8897176

[B24] Tan F. Q. Tu H. Liang W. J. Long J. M. Wu X. M. Zhang H. Y. (2015). Comparative metabolic and transcriptional analysis of a doubled diploid and its diploid citrus rootstock (*C. junos* cv. Ziyang xiangcheng) suggests its potential value for stress resistance improvement. *BMC Plant Biol.* 15:89. 10.1186/s12870-015-0450-4 25848687 PMC4374211

[B25] Xie R. Pan X. Zhang J. Ma Y. He S. Zheng Y. (2018). Effect of salt-stress on gene expression in citrus roots revealed by RNA-seq. *Funct. Integr. Genomics* 18 155–73. 10.1007/s10142-017-0582-8 29264749

[B26] Xie R. Zhang J. Ma Y. Pan X. Dong C. Pang S. (2017). Combined analysis of mRNA and miRNA identifies dehydration and salinity responsive key molecular players in citrus roots. *Sci. Rep.* 7:42094. 10.1038/srep42094 28165059 PMC5292693

[B27] Yacoubi I. Gadaleta A. Mathlouthi N. (2022). Abscisic acid-stress-ripening genes involved in plant response to high salinity and water deficit in durum and common wheat. *Front. Plant Sci.* 13:789701. 10.3389/fpls.2022.789701 35283900 PMC8905601

[B28] Yoshida S. Cui S. Ichihashi Y. Shirasu K. (2016). The Haustorium, a specialized invasive organ in parasitic plants. *Annu. Rev. Plant Biol.* 67 643–67. 10.1146/annurev-arplant-043015-111702 27128469

[B29] Zhang H. Zhu J. Gong Z. Zhu J. (2022). Abiotic stress responses in plants. *Nat. Rev. Genet.* 23 104–19. 10.1038/s41576-021-00413-0 34561623

[B30] Zhang J. Xu Y. Xie J. Zhuang H. Liu H. Shen G. (2021). Parasite dodder enables transfer of bidirectional systemic nitrogen signals between host plants. *Plant Physiol.* 185 1395–410. 10.1093/plphys/kiaa004 33793912 PMC8133666

[B31] Zhou L. J. Gabriel D. W. Duan Y. P. Halbert S. E. Dixon W. N. (2007). First report of dodder transmission of Huanglongbing from naturally infected murraya paniculata to citrus. *Plant Dis.* 91:227. 10.1094/PDIS-91-2-0227B 30781013

