# Supplementary material for: Dodder-transmitted mobile systemic signals activate a salt-stress response characterized by a transcriptome change in Citrus sinensis
Source: Front Plant Sci. 2022 Aug 9;13:986365. doi: 10.3389/fpls.2022.986365 (PMC9422749; doi:10.3389/fpls.2022.986365)
Supplement: Supplementary file 1 [file Presentation_1.pptx]

## Slide 1
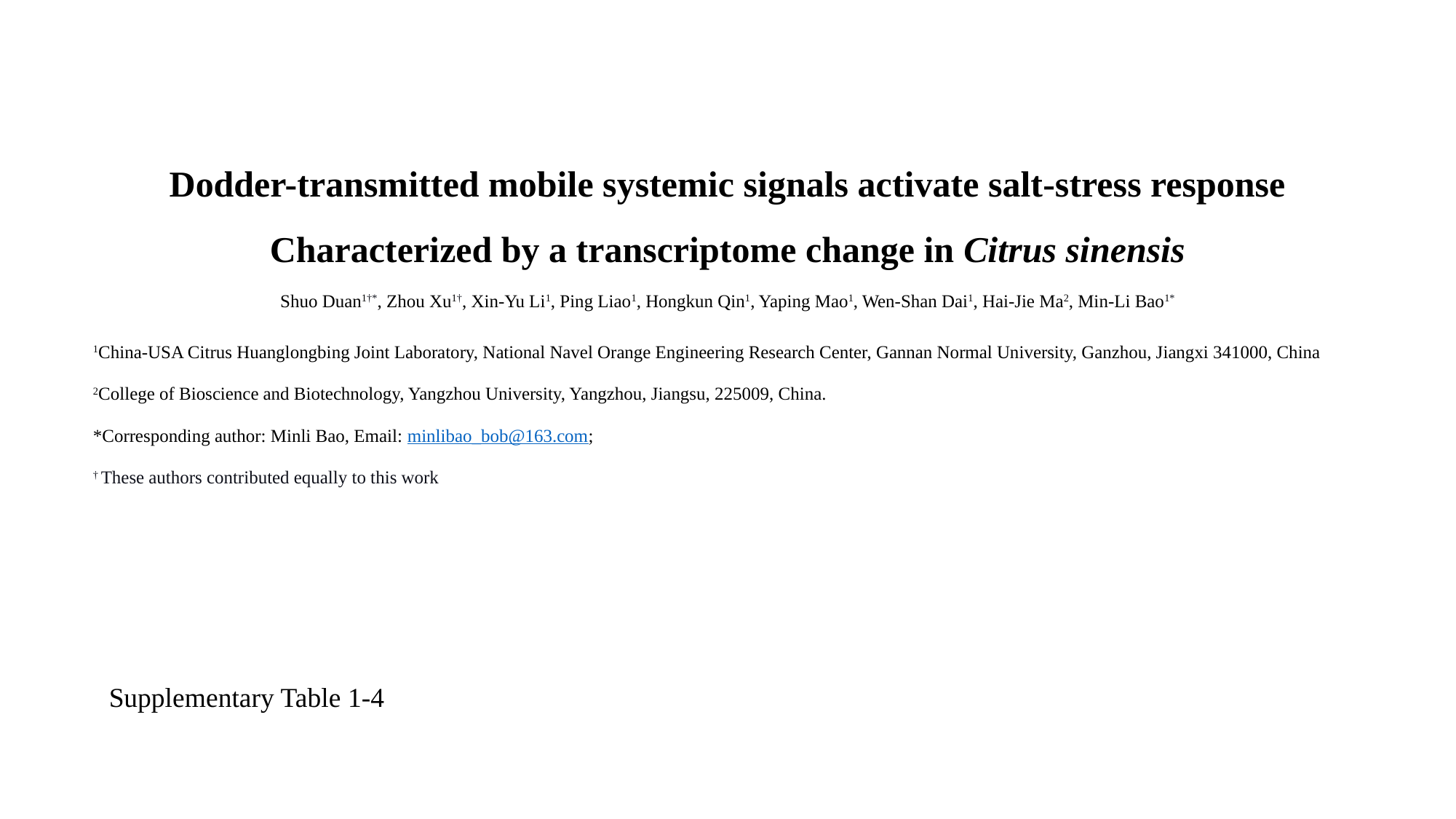

Dodder-transmitted mobile systemic signals activate salt-stress response Characterized by a transcriptome change in Citrus sinensis
Shuo Duan1†*, Zhou Xu1†, Xin-Yu Li1, Ping Liao1, Hongkun Qin1, Yaping Mao1, Wen-Shan Dai1, Hai-Jie Ma2, Min-Li Bao1*
1China-USA Citrus Huanglongbing Joint Laboratory, National Navel Orange Engineering Research Center, Gannan Normal University, Ganzhou, Jiangxi 341000, China
2College of Bioscience and Biotechnology, Yangzhou University, Yangzhou, Jiangsu, 225009, China.
*Corresponding author: Minli Bao, Email: minlibao_bob@163.com;
† These authors contributed equally to this work
Supplementary Table 1-4

## Slide 2
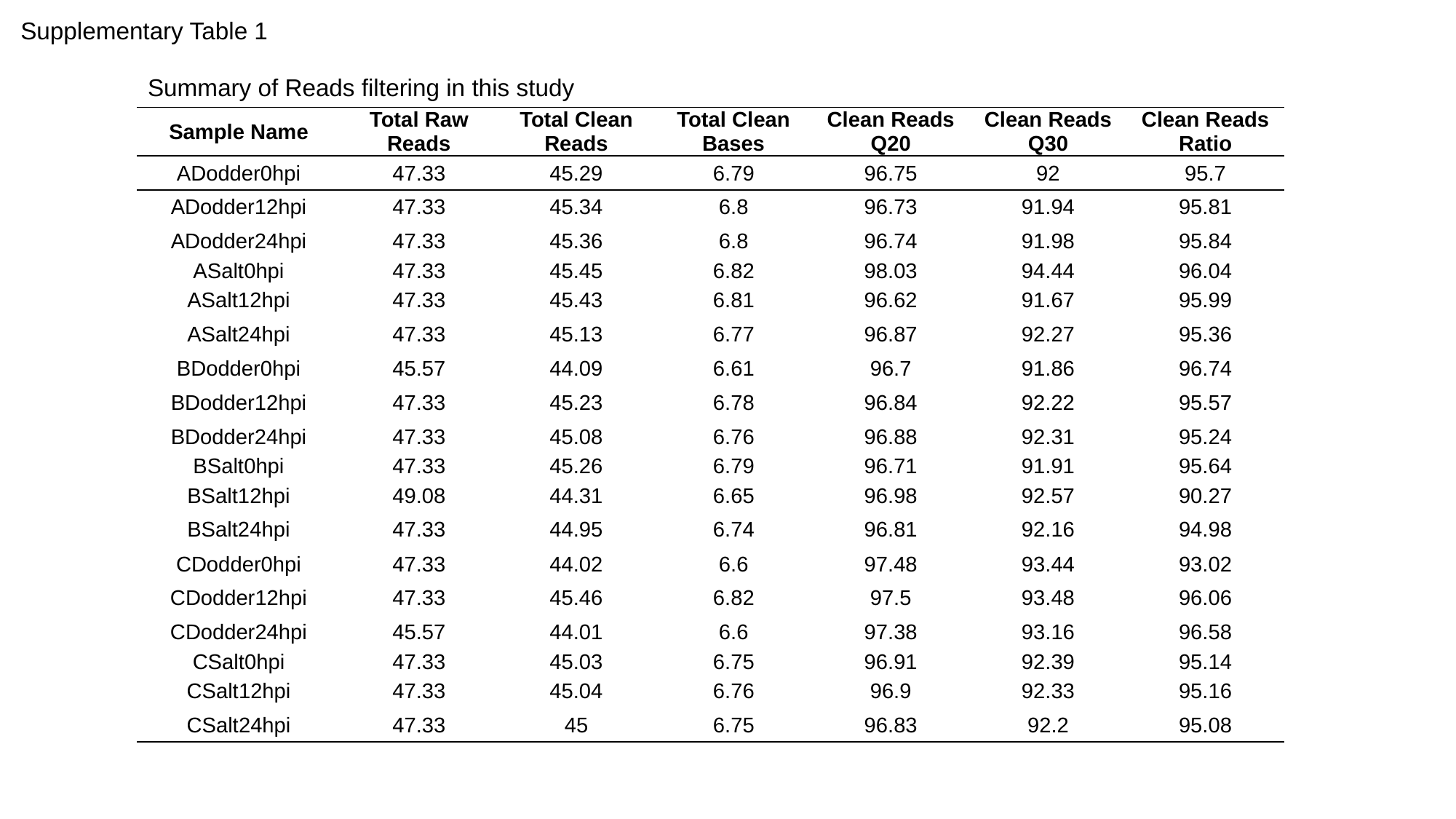

Supplementary Table 1
Summary of Reads filtering in this study
| Sample Name | Total Raw Reads | Total Clean Reads | Total Clean Bases | Clean Reads Q20 | Clean Reads Q30 | Clean Reads Ratio |
| --- | --- | --- | --- | --- | --- | --- |
| ADodder0hpi | 47.33 | 45.29 | 6.79 | 96.75 | 92 | 95.7 |
| ADodder12hpi | 47.33 | 45.34 | 6.8 | 96.73 | 91.94 | 95.81 |
| ADodder24hpi | 47.33 | 45.36 | 6.8 | 96.74 | 91.98 | 95.84 |
| ASalt0hpi | 47.33 | 45.45 | 6.82 | 98.03 | 94.44 | 96.04 |
| ASalt12hpi | 47.33 | 45.43 | 6.81 | 96.62 | 91.67 | 95.99 |
| ASalt24hpi | 47.33 | 45.13 | 6.77 | 96.87 | 92.27 | 95.36 |
| BDodder0hpi | 45.57 | 44.09 | 6.61 | 96.7 | 91.86 | 96.74 |
| BDodder12hpi | 47.33 | 45.23 | 6.78 | 96.84 | 92.22 | 95.57 |
| BDodder24hpi | 47.33 | 45.08 | 6.76 | 96.88 | 92.31 | 95.24 |
| BSalt0hpi | 47.33 | 45.26 | 6.79 | 96.71 | 91.91 | 95.64 |
| BSalt12hpi | 49.08 | 44.31 | 6.65 | 96.98 | 92.57 | 90.27 |
| BSalt24hpi | 47.33 | 44.95 | 6.74 | 96.81 | 92.16 | 94.98 |
| CDodder0hpi | 47.33 | 44.02 | 6.6 | 97.48 | 93.44 | 93.02 |
| CDodder12hpi | 47.33 | 45.46 | 6.82 | 97.5 | 93.48 | 96.06 |
| CDodder24hpi | 45.57 | 44.01 | 6.6 | 97.38 | 93.16 | 96.58 |
| CSalt0hpi | 47.33 | 45.03 | 6.75 | 96.91 | 92.39 | 95.14 |
| CSalt12hpi | 47.33 | 45.04 | 6.76 | 96.9 | 92.33 | 95.16 |
| CSalt24hpi | 47.33 | 45 | 6.75 | 96.83 | 92.2 | 95.08 |

## Slide 3
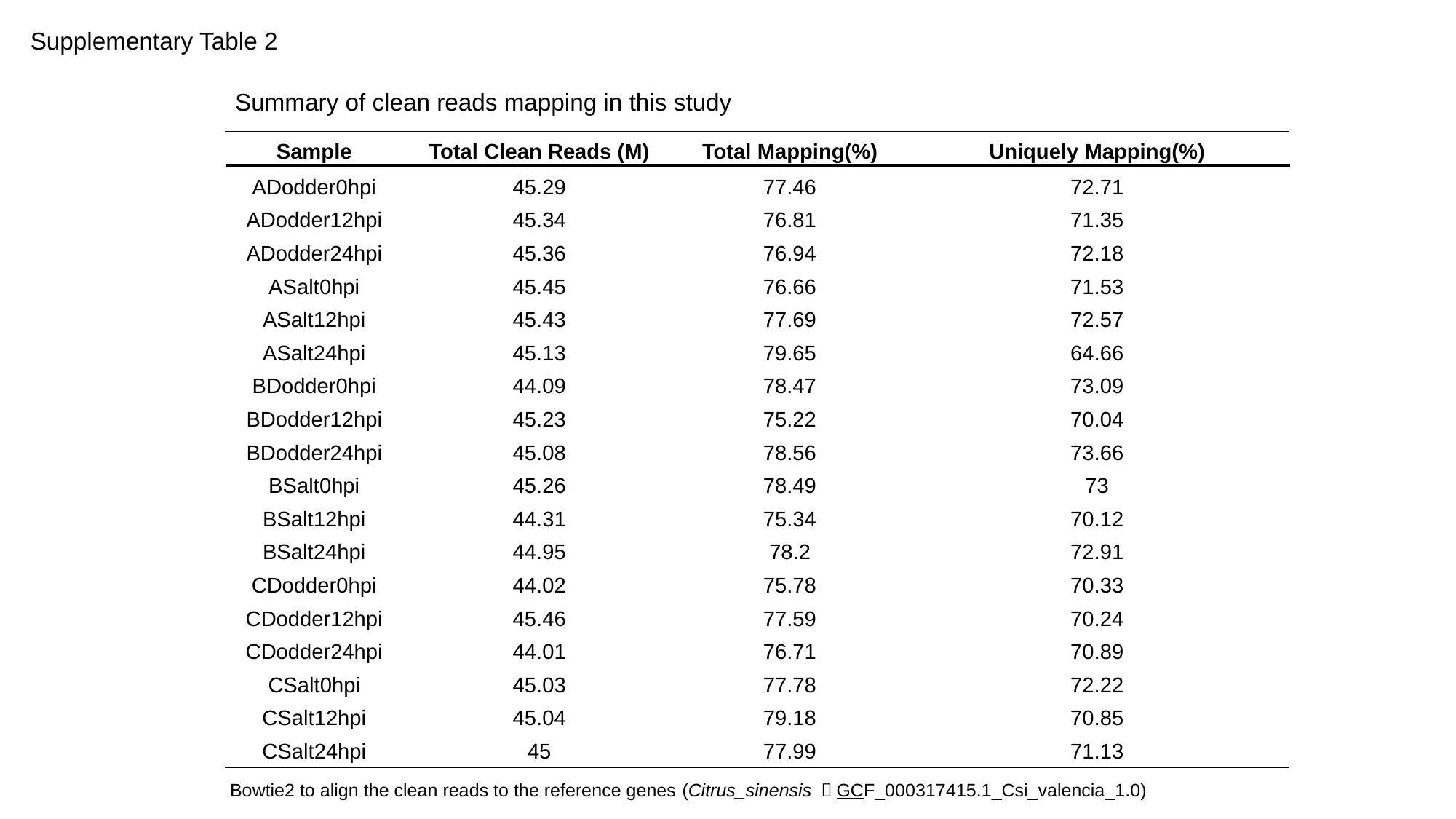

Supplementary Table 2
Summary of clean reads mapping in this study
| Sample | Total Clean Reads (M) | Total Mapping(%) | Uniquely Mapping(%) |
| --- | --- | --- | --- |
| ADodder0hpi | 45.29 | 77.46 | 72.71 |
| ADodder12hpi | 45.34 | 76.81 | 71.35 |
| ADodder24hpi | 45.36 | 76.94 | 72.18 |
| ASalt0hpi | 45.45 | 76.66 | 71.53 |
| ASalt12hpi | 45.43 | 77.69 | 72.57 |
| ASalt24hpi | 45.13 | 79.65 | 64.66 |
| BDodder0hpi | 44.09 | 78.47 | 73.09 |
| BDodder12hpi | 45.23 | 75.22 | 70.04 |
| BDodder24hpi | 45.08 | 78.56 | 73.66 |
| BSalt0hpi | 45.26 | 78.49 | 73 |
| BSalt12hpi | 44.31 | 75.34 | 70.12 |
| BSalt24hpi | 44.95 | 78.2 | 72.91 |
| CDodder0hpi | 44.02 | 75.78 | 70.33 |
| CDodder12hpi | 45.46 | 77.59 | 70.24 |
| CDodder24hpi | 44.01 | 76.71 | 70.89 |
| CSalt0hpi | 45.03 | 77.78 | 72.22 |
| CSalt12hpi | 45.04 | 79.18 | 70.85 |
| CSalt24hpi | 45 | 77.99 | 71.13 |
 Bowtie2 to align the clean reads to the reference genes (Citrus_sinensis ：GCF_000317415.1_Csi_valencia_1.0)

## Slide 4
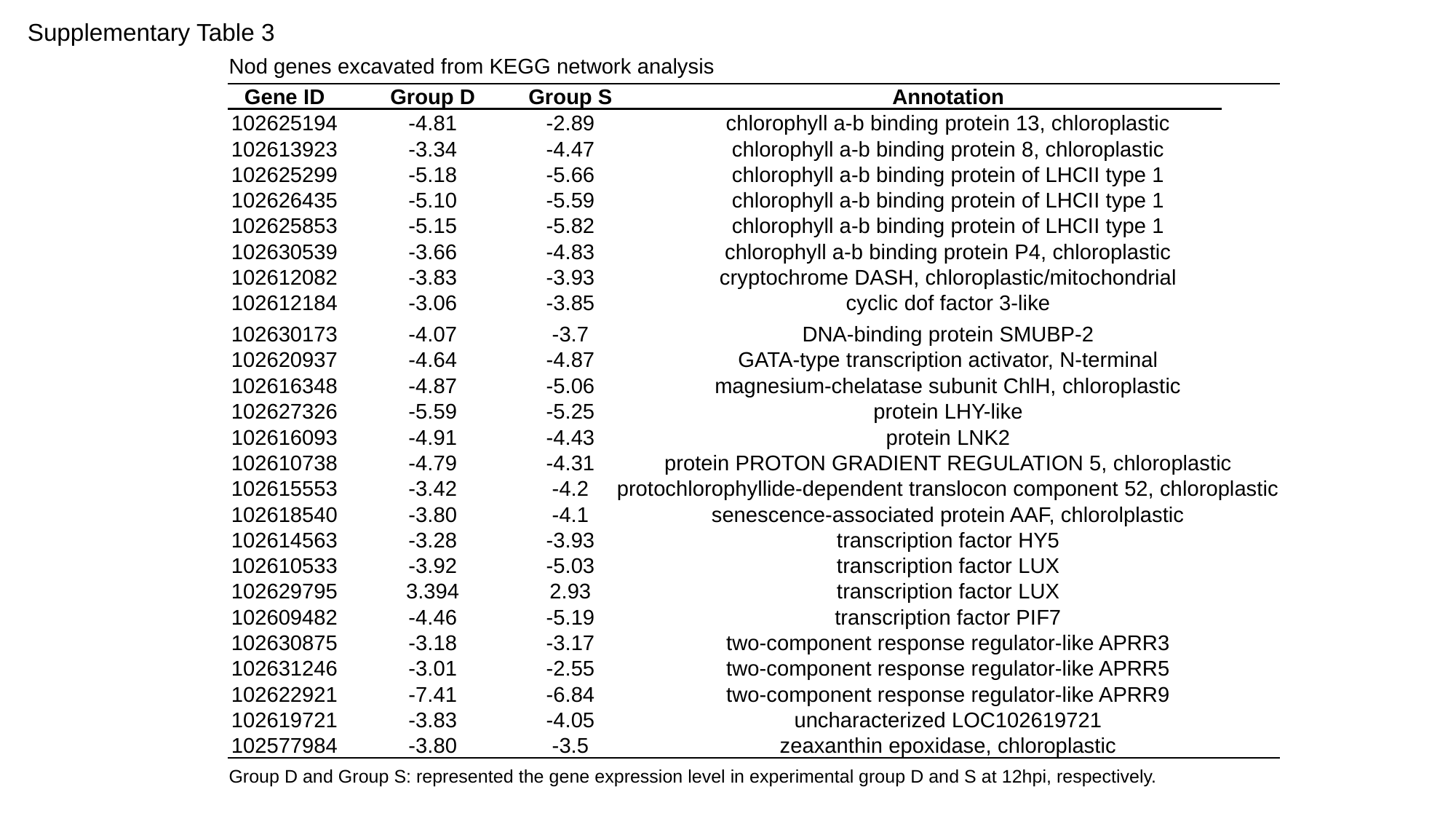

Supplementary Table 3
Nod genes excavated from KEGG network analysis
| Gene ID | Group D | Group S | Annotation |
| --- | --- | --- | --- |
| 102625194 | -4.81 | -2.89 | chlorophyll a-b binding protein 13, chloroplastic |
| 102613923 | -3.34 | -4.47 | chlorophyll a-b binding protein 8, chloroplastic |
| 102625299 | -5.18 | -5.66 | chlorophyll a-b binding protein of LHCII type 1 |
| 102626435 | -5.10 | -5.59 | chlorophyll a-b binding protein of LHCII type 1 |
| 102625853 | -5.15 | -5.82 | chlorophyll a-b binding protein of LHCII type 1 |
| 102630539 | -3.66 | -4.83 | chlorophyll a-b binding protein P4, chloroplastic |
| 102612082 | -3.83 | -3.93 | cryptochrome DASH, chloroplastic/mitochondrial |
| 102612184 | -3.06 | -3.85 | cyclic dof factor 3-like |
| 102630173 | -4.07 | -3.7 | DNA-binding protein SMUBP-2 |
| 102620937 | -4.64 | -4.87 | GATA-type transcription activator, N-terminal |
| 102616348 | -4.87 | -5.06 | magnesium-chelatase subunit ChlH, chloroplastic |
| 102627326 | -5.59 | -5.25 | protein LHY-like |
| 102616093 | -4.91 | -4.43 | protein LNK2 |
| 102610738 | -4.79 | -4.31 | protein PROTON GRADIENT REGULATION 5, chloroplastic |
| 102615553 | -3.42 | -4.2 | protochlorophyllide-dependent translocon component 52, chloroplastic |
| 102618540 | -3.80 | -4.1 | senescence-associated protein AAF, chlorolplastic |
| 102614563 | -3.28 | -3.93 | transcription factor HY5 |
| 102610533 | -3.92 | -5.03 | transcription factor LUX |
| 102629795 | 3.394 | 2.93 | transcription factor LUX |
| 102609482 | -4.46 | -5.19 | transcription factor PIF7 |
| 102630875 | -3.18 | -3.17 | two-component response regulator-like APRR3 |
| 102631246 | -3.01 | -2.55 | two-component response regulator-like APRR5 |
| 102622921 | -7.41 | -6.84 | two-component response regulator-like APRR9 |
| 102619721 | -3.83 | -4.05 | uncharacterized LOC102619721 |
| 102577984 | -3.80 | -3.5 | zeaxanthin epoxidase, chloroplastic |
Group D and Group S: represented the gene expression level in experimental group D and S at 12hpi, respectively.

## Slide 5
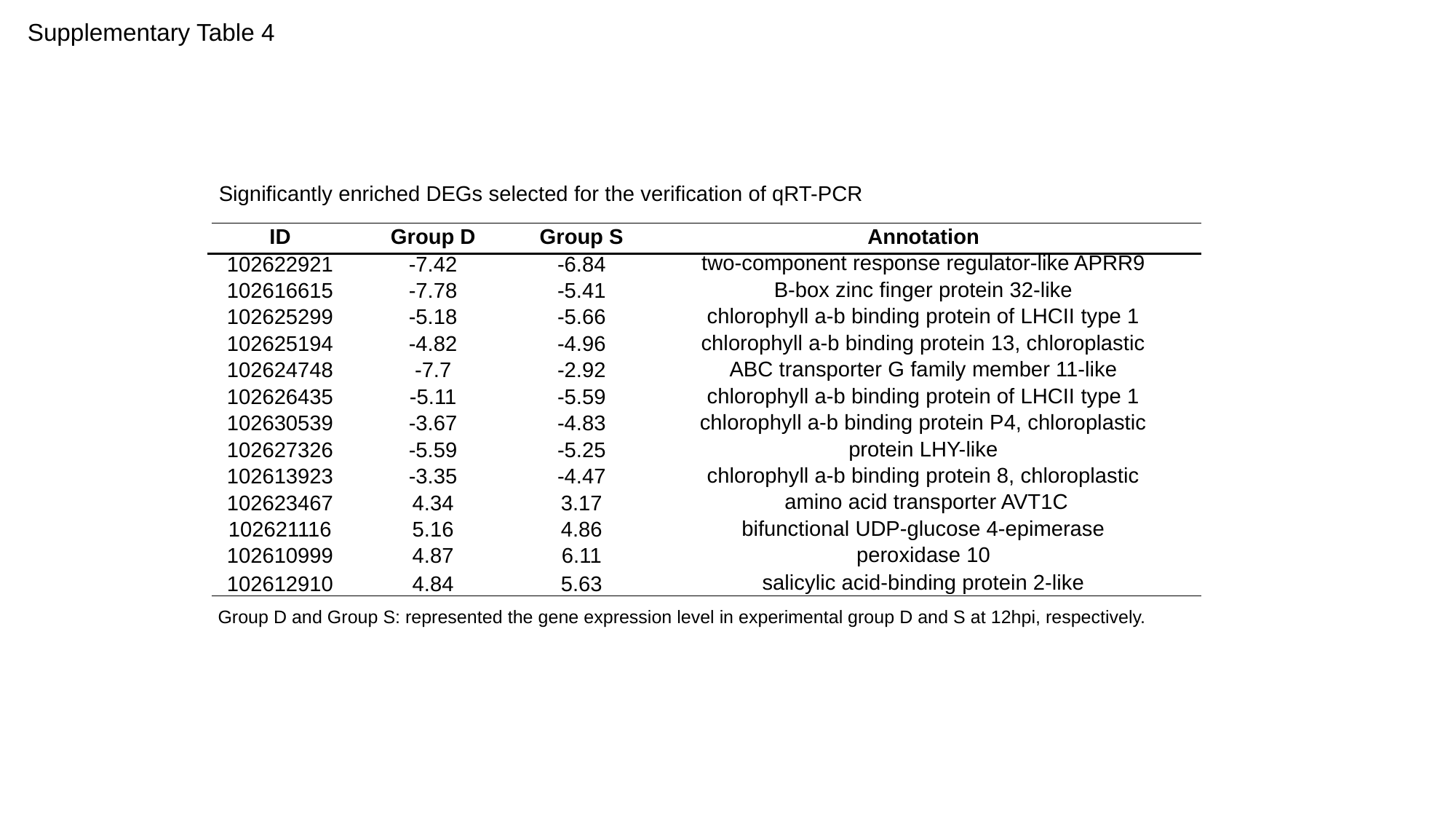

Supplementary Table 4
Significantly enriched DEGs selected for the verification of qRT-PCR
| ID | Group D | Group S | Annotation |
| --- | --- | --- | --- |
| 102622921 | -7.42 | -6.84 | two-component response regulator-like APRR9 |
| 102616615 | -7.78 | -5.41 | B-box zinc finger protein 32-like |
| 102625299 | -5.18 | -5.66 | chlorophyll a-b binding protein of LHCII type 1 |
| 102625194 | -4.82 | -4.96 | chlorophyll a-b binding protein 13, chloroplastic |
| 102624748 | -7.7 | -2.92 | ABC transporter G family member 11-like |
| 102626435 | -5.11 | -5.59 | chlorophyll a-b binding protein of LHCII type 1 |
| 102630539 | -3.67 | -4.83 | chlorophyll a-b binding protein P4, chloroplastic |
| 102627326 | -5.59 | -5.25 | protein LHY-like |
| 102613923 | -3.35 | -4.47 | chlorophyll a-b binding protein 8, chloroplastic |
| 102623467 | 4.34 | 3.17 | amino acid transporter AVT1C |
| 102621116 | 5.16 | 4.86 | bifunctional UDP-glucose 4-epimerase |
| 102610999 | 4.87 | 6.11 | peroxidase 10 |
| 102612910 | 4.84 | 5.63 | salicylic acid-binding protein 2-like |
Group D and Group S: represented the gene expression level in experimental group D and S at 12hpi, respectively.

## Slide 6
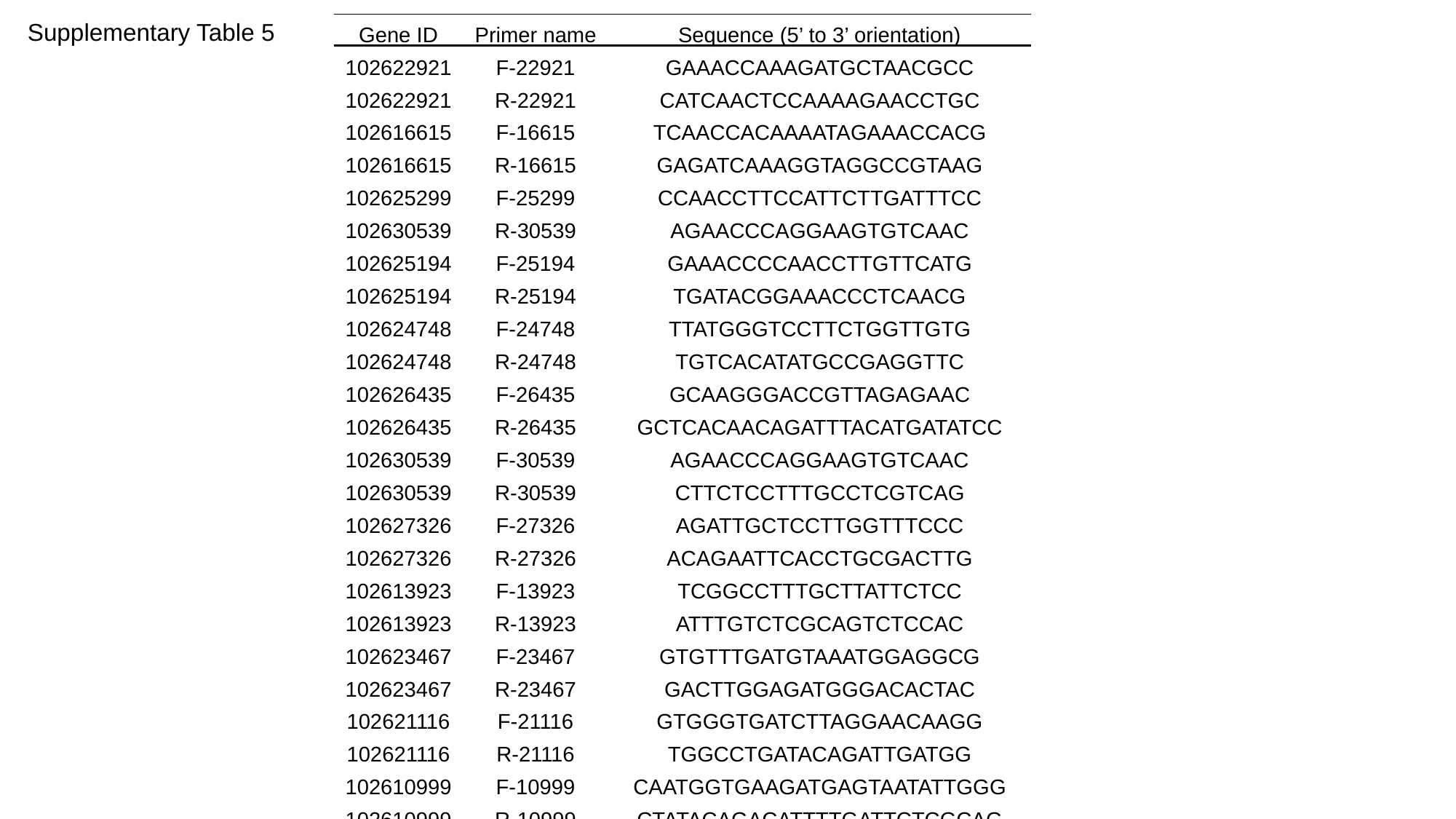

Supplementary Table 5
| Gene ID | Primer name | Sequence (5’ to 3’ orientation) |
| --- | --- | --- |
| 102622921 | F-22921 | GAAACCAAAGATGCTAACGCC |
| 102622921 | R-22921 | CATCAACTCCAAAAGAACCTGC |
| 102616615 | F-16615 | TCAACCACAAAATAGAAACCACG |
| 102616615 | R-16615 | GAGATCAAAGGTAGGCCGTAAG |
| 102625299 | F-25299 | CCAACCTTCCATTCTTGATTTCC |
| 102630539 | R-30539 | AGAACCCAGGAAGTGTCAAC |
| 102625194 | F-25194 | GAAACCCCAACCTTGTTCATG |
| 102625194 | R-25194 | TGATACGGAAACCCTCAACG |
| 102624748 | F-24748 | TTATGGGTCCTTCTGGTTGTG |
| 102624748 | R-24748 | TGTCACATATGCCGAGGTTC |
| 102626435 | F-26435 | GCAAGGGACCGTTAGAGAAC |
| 102626435 | R-26435 | GCTCACAACAGATTTACATGATATCC |
| 102630539 | F-30539 | AGAACCCAGGAAGTGTCAAC |
| 102630539 | R-30539 | CTTCTCCTTTGCCTCGTCAG |
| 102627326 | F-27326 | AGATTGCTCCTTGGTTTCCC |
| 102627326 | R-27326 | ACAGAATTCACCTGCGACTTG |
| 102613923 | F-13923 | TCGGCCTTTGCTTATTCTCC |
| 102613923 | R-13923 | ATTTGTCTCGCAGTCTCCAC |
| 102623467 | F-23467 | GTGTTTGATGTAAATGGAGGCG |
| 102623467 | R-23467 | GACTTGGAGATGGGACACTAC |
| 102621116 | F-21116 | GTGGGTGATCTTAGGAACAAGG |
| 102621116 | R-21116 | TGGCCTGATACAGATTGATGG |
| 102610999 | F-10999 | CAATGGTGAAGATGAGTAATATTGGG |
| 102610999 | R-10999 | CTATACAGACATTTTGATTCTCGCAG |
| 102612910 | F-12910 | CGTGGCTGTTTTCGTAACTG |
| 102612910 | R-12910 | CCGTCCTCTTTTCCCATCTTC |
| 102614495 | F-14495 | CTTGTTTCGGTCGACTTCAG |
| 102614495 | R-14495 | GTTGTCGTACCATGCAATCA |
