# Supplementary material for: Dodder-transmitted mobile systemic signals activate a salt-stress response characterized by a transcriptome change in Citrus sinensis
Source: Front Plant Sci. 2022 Aug 9;13:986365. doi: 10.3389/fpls.2022.986365 (PMC9422749; doi:10.3389/fpls.2022.986365)
Supplement: Supplementary file 2 [file Presentation_2.PPTX]

## Slide 1
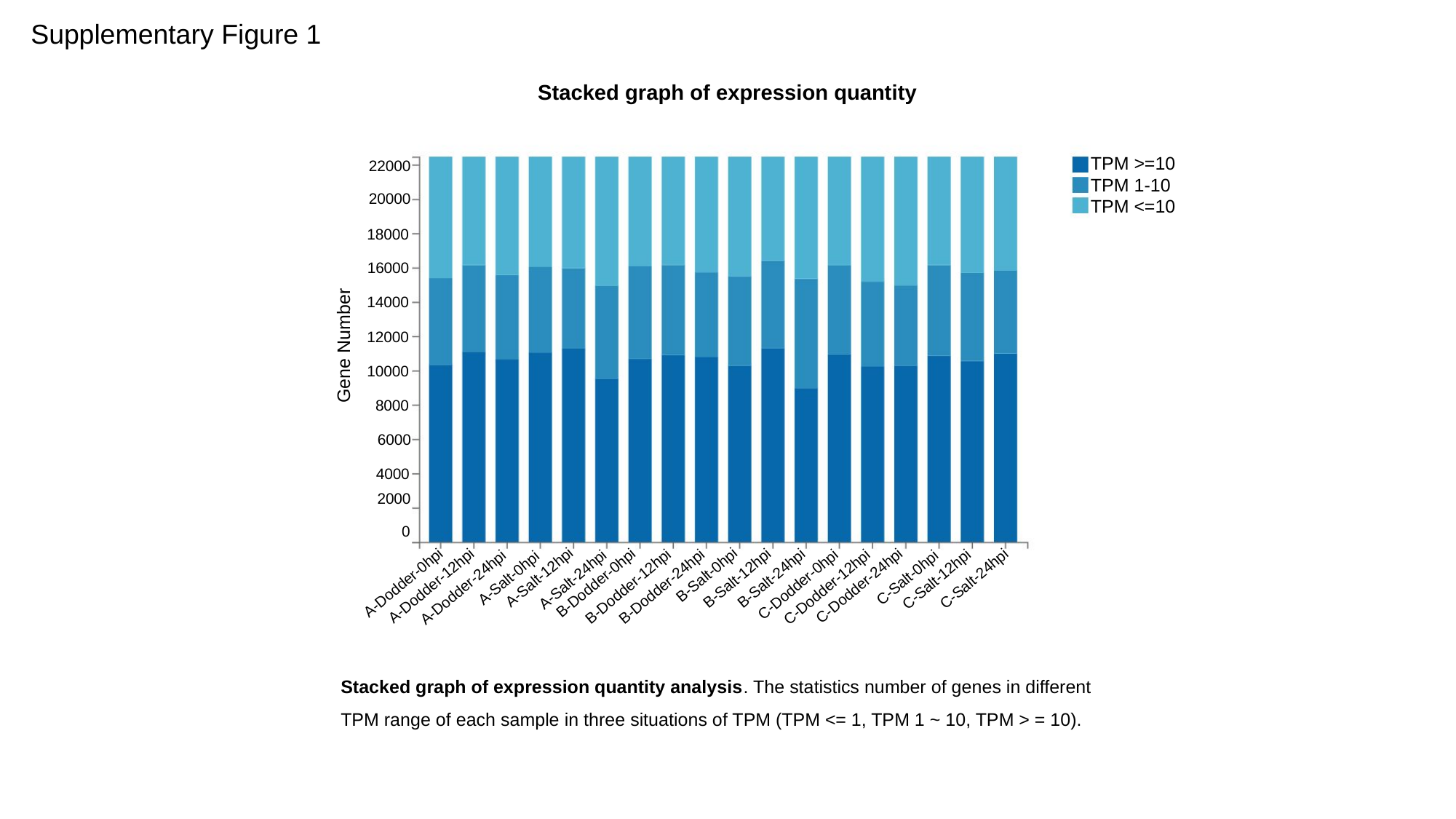

Supplementary Figure 1
Stacked graph of expression quantity
TPM >=10
22000
TPM 1-10
20000
TPM <=10
18000
16000
Gene Number
14000
12000
10000
8000
6000
4000
2000
0
B-Salt-0hpi
B-Salt-12hpi
C-Salt-0hpi
A-Salt-0hpi
B-Salt-24hpi
A-Salt-12hpi
A-Salt-24hpi
C-Salt-24hpi
C-Salt-12hpi
A-Dodder-0hpi
B-Dodder-0hpi
C-Dodder-0hpi
A-Dodder-12hpi
C-Dodder-24hpi
B-Dodder-12hpi
B-Dodder-24hpi
C-Dodder-12hpi
A-Dodder-24hpi
Stacked graph of expression quantity analysis. The statistics number of genes in different TPM range of each sample in three situations of TPM (TPM <= 1, TPM 1 ~ 10, TPM > = 10).

## Slide 2
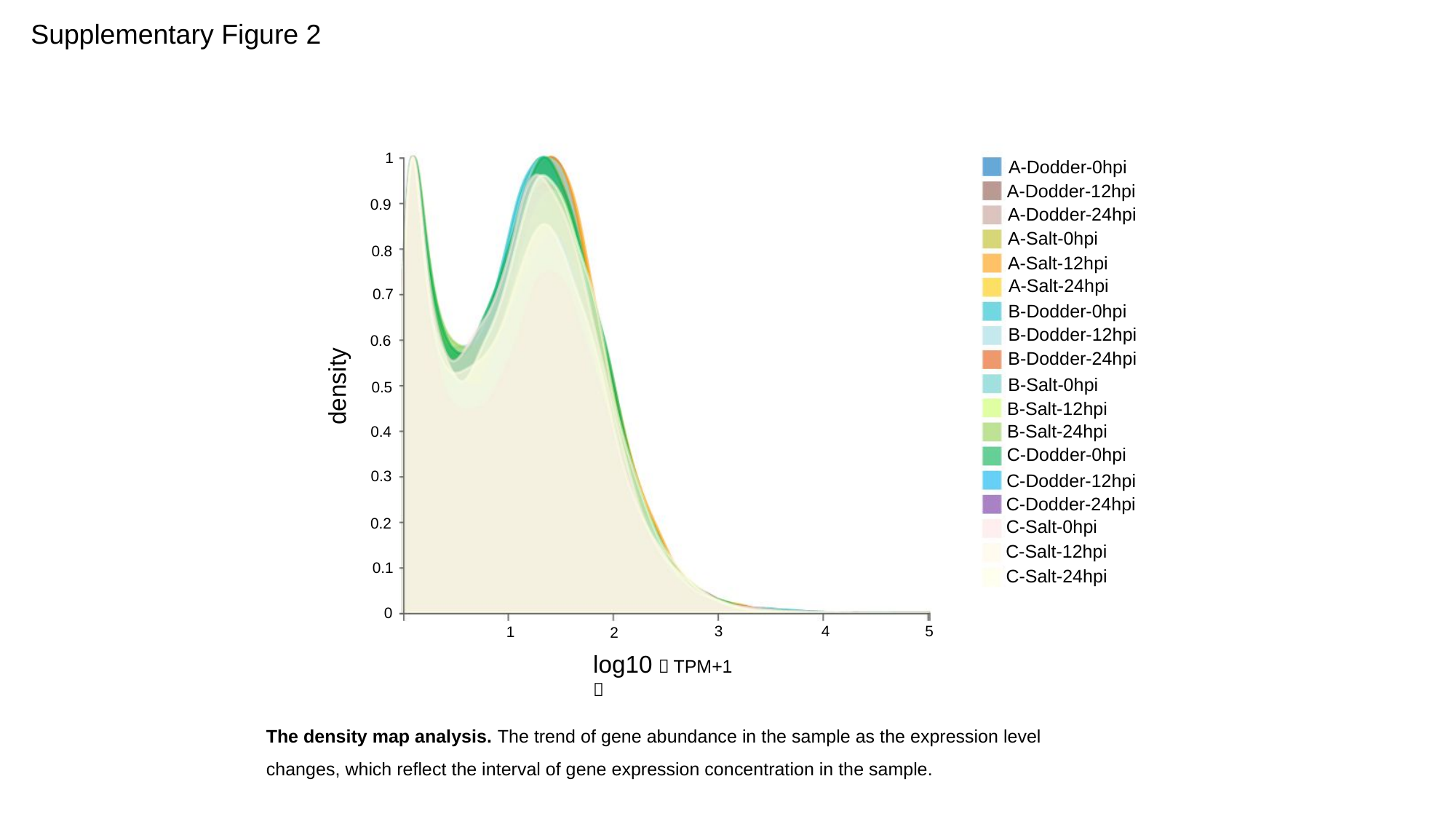

Supplementary Figure 2
1
A-Dodder-0hpi
A-Dodder-12hpi
0.9
A-Dodder-24hpi
A-Salt-0hpi
0.8
A-Salt-12hpi
A-Salt-24hpi
0.7
B-Dodder-0hpi
B-Dodder-12hpi
0.6
density
B-Dodder-24hpi
B-Salt-0hpi
0.5
B-Salt-12hpi
B-Salt-24hpi
0.4
C-Dodder-0hpi
0.3
C-Dodder-12hpi
C-Dodder-24hpi
0.2
C-Salt-0hpi
C-Salt-12hpi
0.1
C-Salt-24hpi
0
3
4
5
1
2
log10（TPM+1）
The density map analysis. The trend of gene abundance in the sample as the expression level changes, which reflect the interval of gene expression concentration in the sample.

## Slide 3
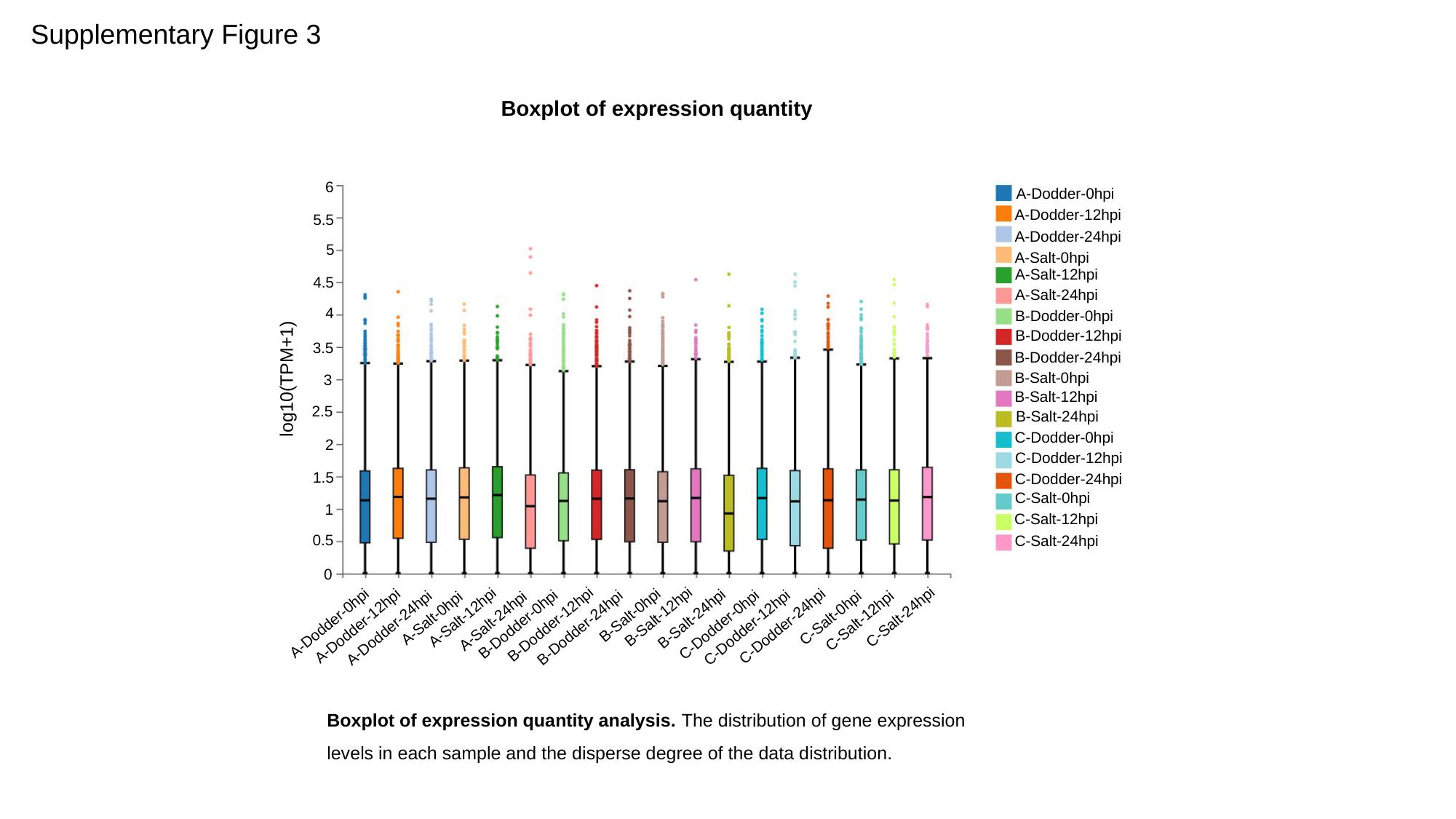

Supplementary Figure 3
Boxplot of expression quantity
6
A-Dodder-0hpi
A-Dodder-12hpi
5.5
A-Dodder-24hpi
5
A-Salt-0hpi
A-Salt-12hpi
4.5
A-Salt-24hpi
4
B-Dodder-0hpi
log10(TPM+1)
B-Dodder-12hpi
3.5
B-Dodder-24hpi
B-Salt-0hpi
3
B-Salt-12hpi
2.5
B-Salt-24hpi
C-Dodder-0hpi
2
C-Dodder-12hpi
1.5
C-Dodder-24hpi
C-Salt-0hpi
1
C-Salt-12hpi
0.5
C-Salt-24hpi
0
B-Salt-0hpi
B-Salt-12hpi
C-Salt-0hpi
C-Salt-24hpi
A-Salt-0hpi
B-Salt-24hpi
A-Salt-12hpi
A-Salt-24hpi
A-Dodder-0hpi
C-Salt-12hpi
B-Dodder-0hpi
C-Dodder-0hpi
B-Dodder-12hpi
A-Dodder-12hpi
C-Dodder-24hpi
C-Dodder-12hpi
B-Dodder-24hpi
A-Dodder-24hpi
Boxplot of expression quantity analysis. The distribution of gene expression levels in each sample and the disperse degree of the data distribution.

## Slide 4
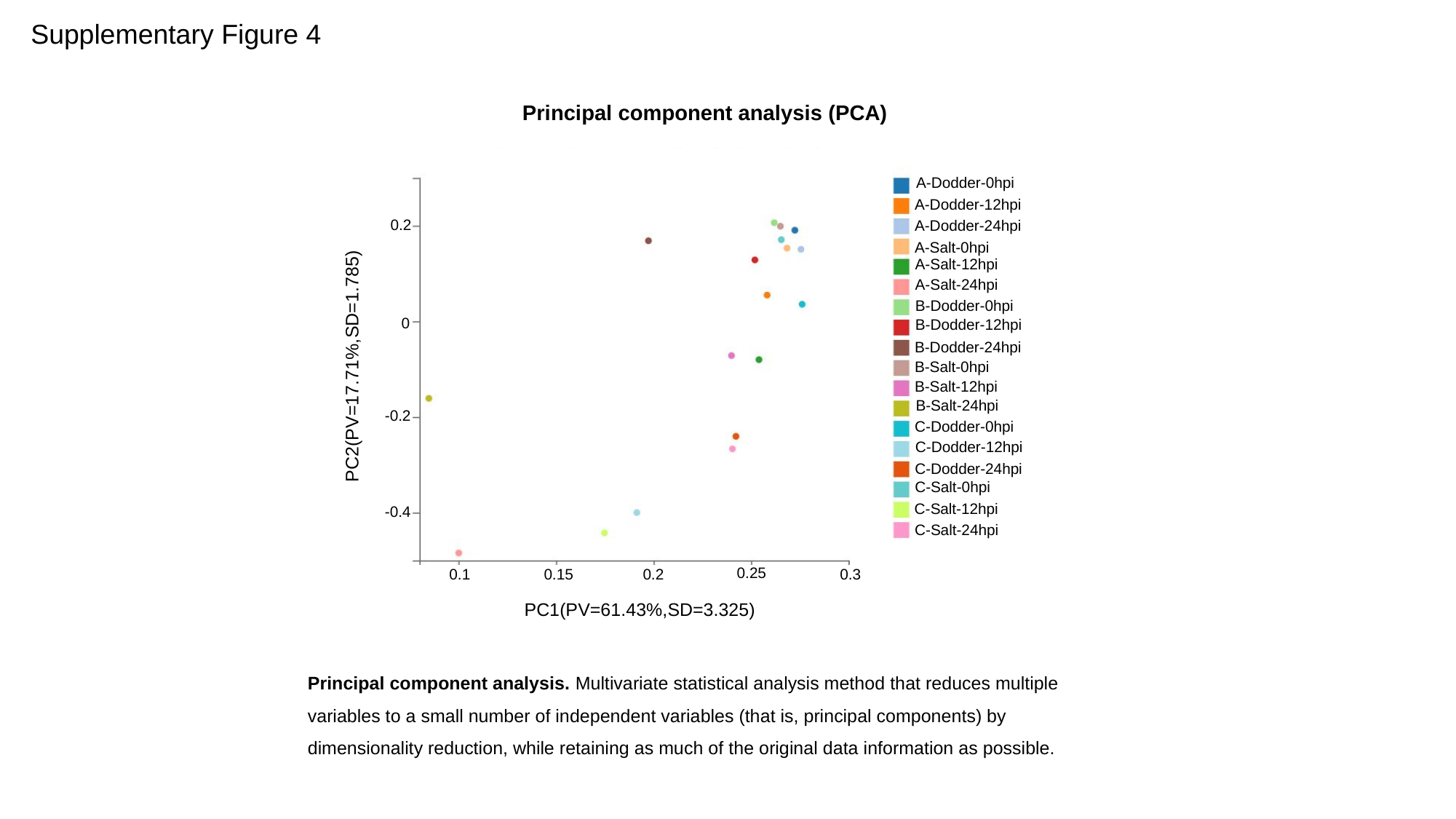

Supplementary Figure 4
Principal component analysis (PCA)
A-Dodder-0hpi
A-Dodder-12hpi
0.2
A-Dodder-24hpi
A-Salt-0hpi
PC2(PV=17.71%,SD=1.785)
A-Salt-12hpi
A-Salt-24hpi
B-Dodder-0hpi
0
B-Dodder-12hpi
B-Dodder-24hpi
B-Salt-0hpi
B-Salt-12hpi
B-Salt-24hpi
-0.2
C-Dodder-0hpi
C-Dodder-12hpi
C-Dodder-24hpi
C-Salt-0hpi
C-Salt-12hpi
-0.4
C-Salt-24hpi
0.25
0.1
0.2
0.3
0.15
PC1(PV=61.43%,SD=3.325)
Principal component analysis. Multivariate statistical analysis method that reduces multiple variables to a small number of independent variables (that is, principal components) by dimensionality reduction, while retaining as much of the original data information as possible.

## Slide 5
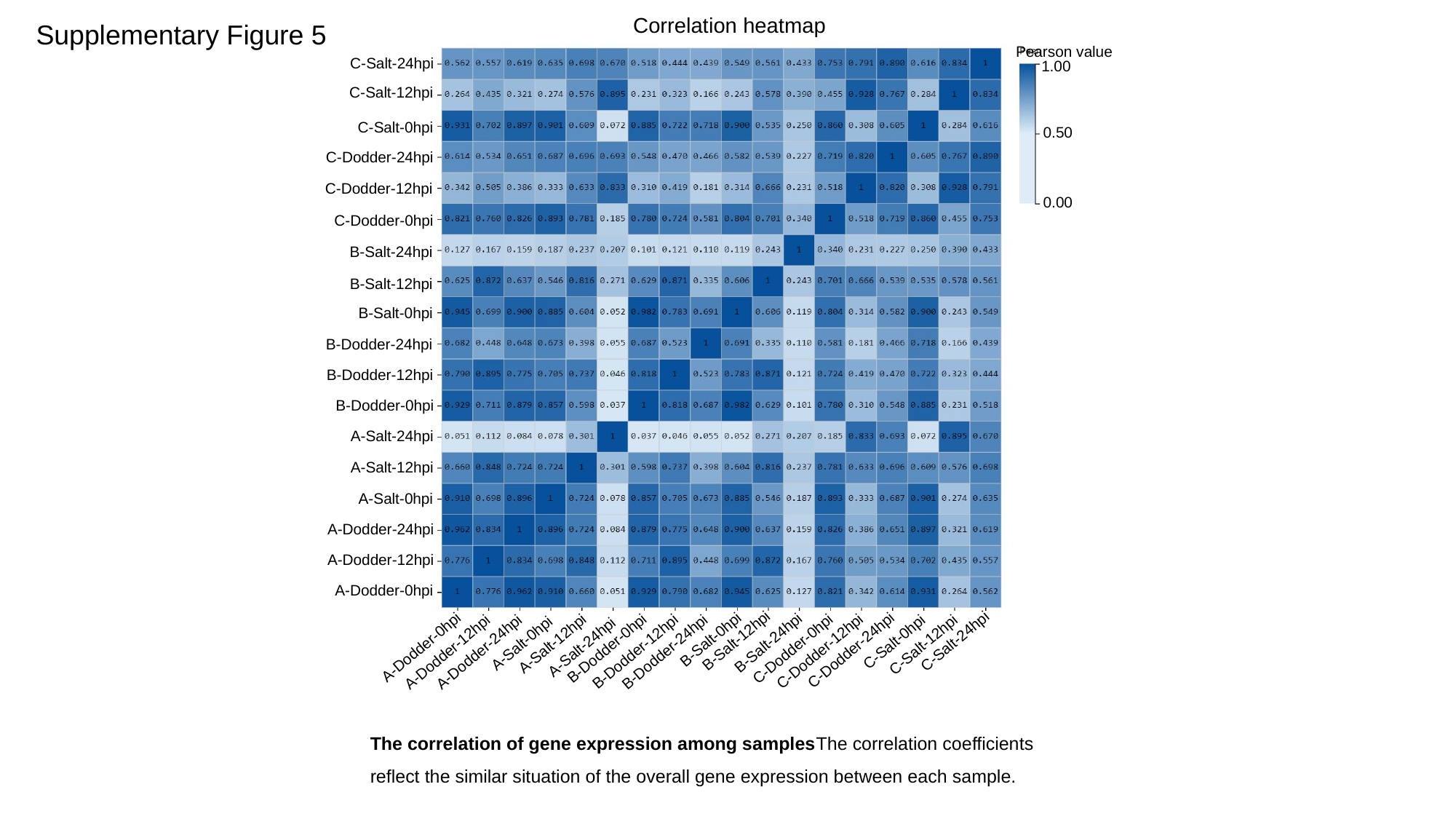

Supplementary Figure 5
Correlation heatmap
Pearson value
C-Salt-24hpi
1.00
C-Salt-12hpi
C-Salt-0hpi
0.50
C-Dodder-24hpi
C-Dodder-12hpi
0.00
C-Dodder-0hpi
B-Salt-24hpi
B-Salt-12hpi
B-Salt-0hpi
B-Dodder-24hpi
B-Dodder-12hpi
B-Dodder-0hpi
A-Salt-24hpi
A-Salt-12hpi
A-Salt-0hpi
A-Dodder-24hpi
A-Dodder-12hpi
A-Dodder-0hpi
B-Salt-0hpi
B-Salt-12hpi
C-Salt-0hpi
C-Salt-24hpi
B-Salt-24hpi
A-Salt-0hpi
A-Salt-12hpi
A-Dodder-0hpi
C-Salt-12hpi
A-Salt-24hpi
B-Dodder-0hpi
C-Dodder-0hpi
C-Dodder-24hpi
C-Dodder-12hpi
B-Dodder-12hpi
B-Dodder-24hpi
A-Dodder-24hpi
A-Dodder-12hpi
The correlation of gene expression among samplesThe correlation coefficients reflect the similar situation of the overall gene expression between each sample.

## Slide 6
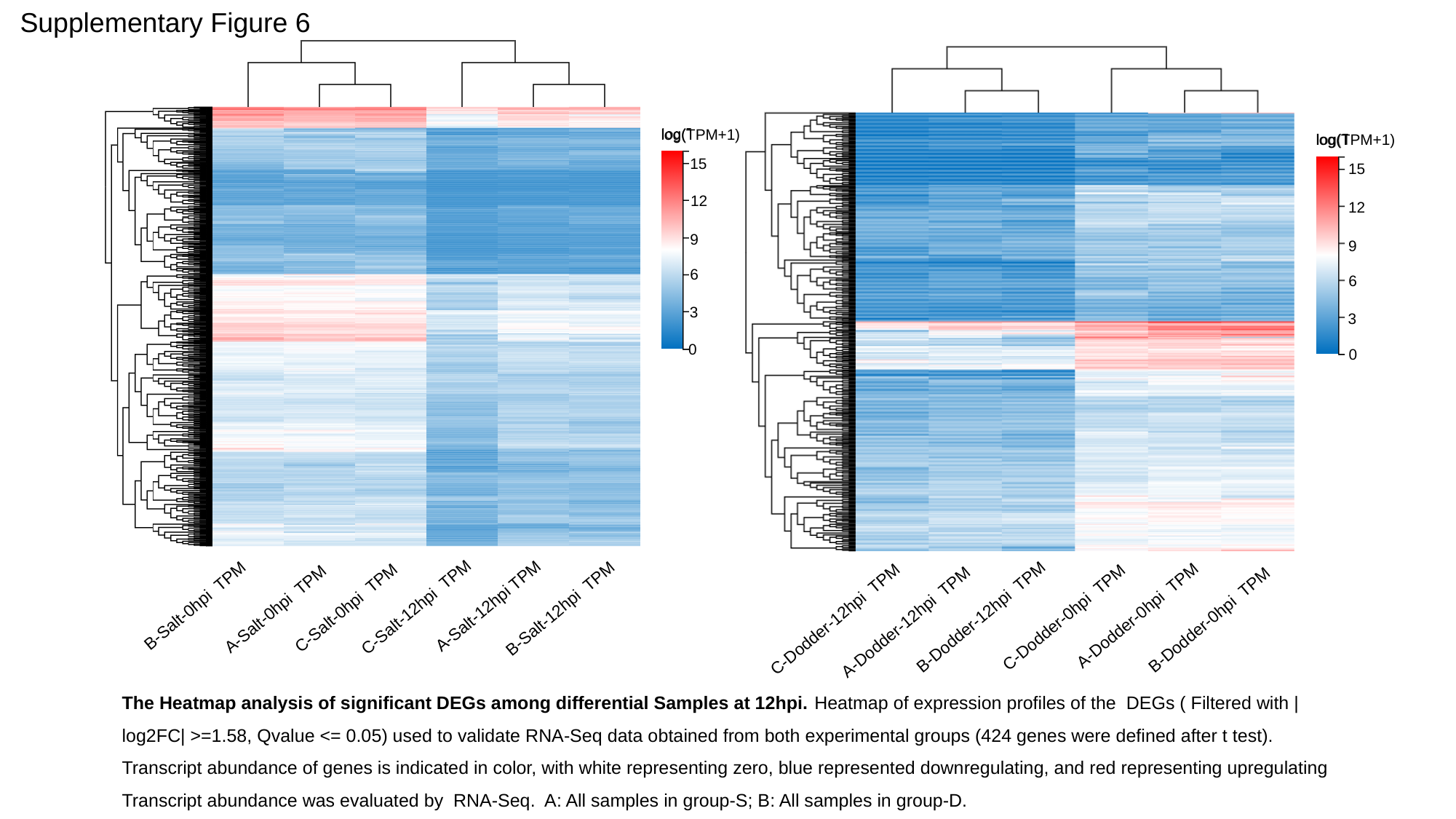

Supplementary Figure 6
log(TPM+1)
log(TPM+1)
15
15
12
12
9
9
6
6
3
3
0
0
C-Salt-12hpi TPM
B-Salt-0hpi TPM
A-Salt-12hpi TPM
C-Salt-0hpi TPM
B-Salt-12hpi TPM
A-Salt-0hpi TPM
A-Dodder-0hpi TPM
B-Dodder-12hpi TPM
C-Dodder-0hpi TPM
B-Dodder-0hpi TPM
C-Dodder-12hpi TPM
A-Dodder-12hpi TPM
The Heatmap analysis of significant DEGs among differential Samples at 12hpi. Heatmap of expression profiles of the DEGs ( Filtered with |log2FC| >=1.58, Qvalue <= 0.05) used to validate RNA-Seq data obtained from both experimental groups (424 genes were defined after t test). Transcript abundance of genes is indicated in color, with white representing zero, blue represented downregulating, and red representing upregulating Transcript abundance was evaluated by RNA-Seq. A: All samples in group-S; B: All samples in group-D.

## Slide 7
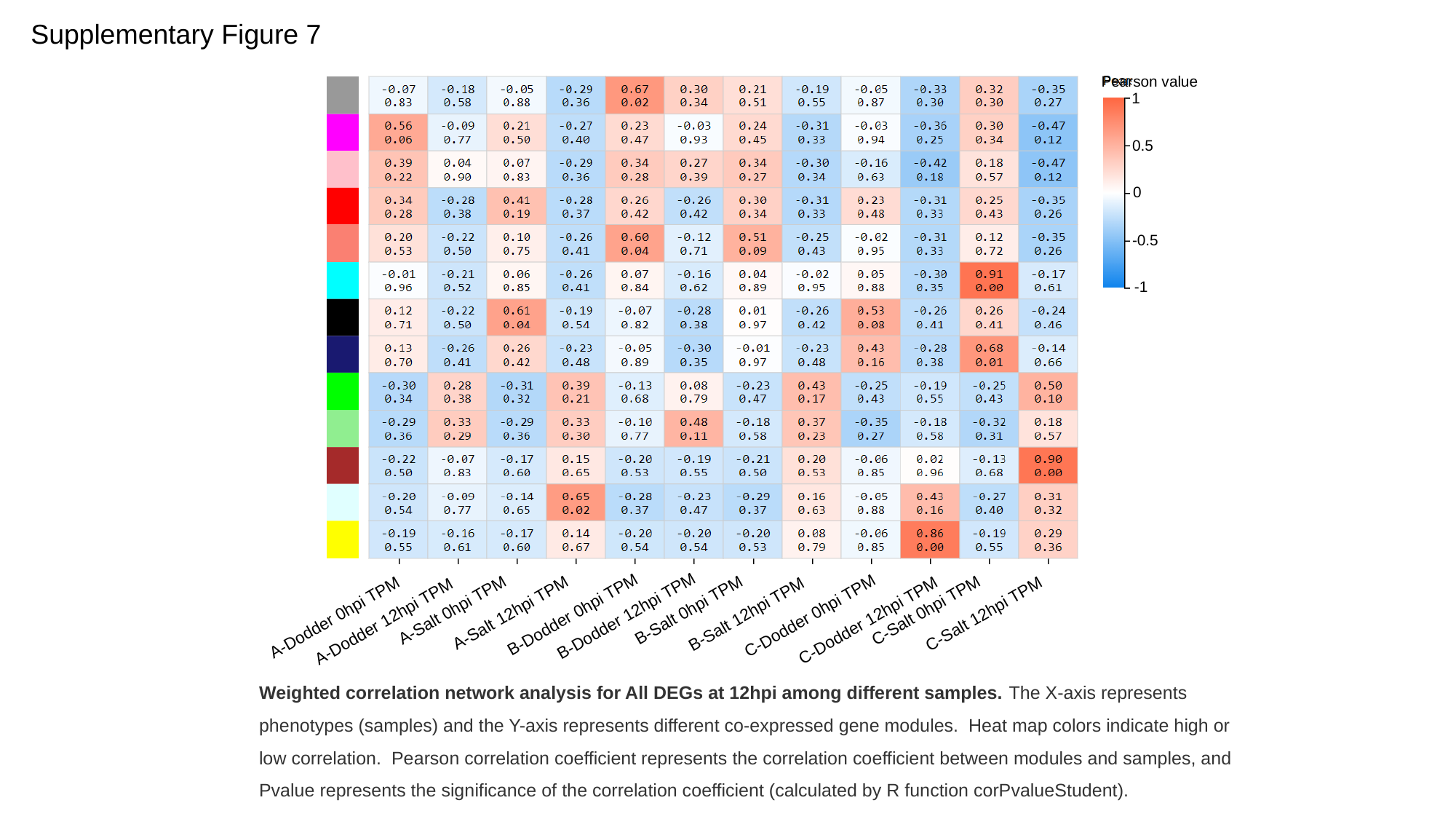

Supplementary Figure 7
Pearson value
A-Salt 0hpi TPM
B-Salt 0hpi TPM
C-Salt 0hpi TPM
A-Salt 12hpi TPM
C-Salt 12hpi TPM
B-Salt 12hpi TPM
B-Dodder 0hpi TPM
C-Dodder 0hpi TPM
B-Dodder 12hpi TPM
A-Dodder 0hpi TPM
C-Dodder 12hpi TPM
A-Dodder 12hpi TPM
1
0.5
0
-0.5
-1
Weighted correlation network analysis for All DEGs at 12hpi among different samples. The X-axis represents phenotypes (samples) and the Y-axis represents different co-expressed gene modules.  Heat map colors indicate high or low correlation.  Pearson correlation coefficient represents the correlation coefficient between modules and samples, and Pvalue represents the significance of the correlation coefficient (calculated by R function corPvalueStudent).

## Slide 8
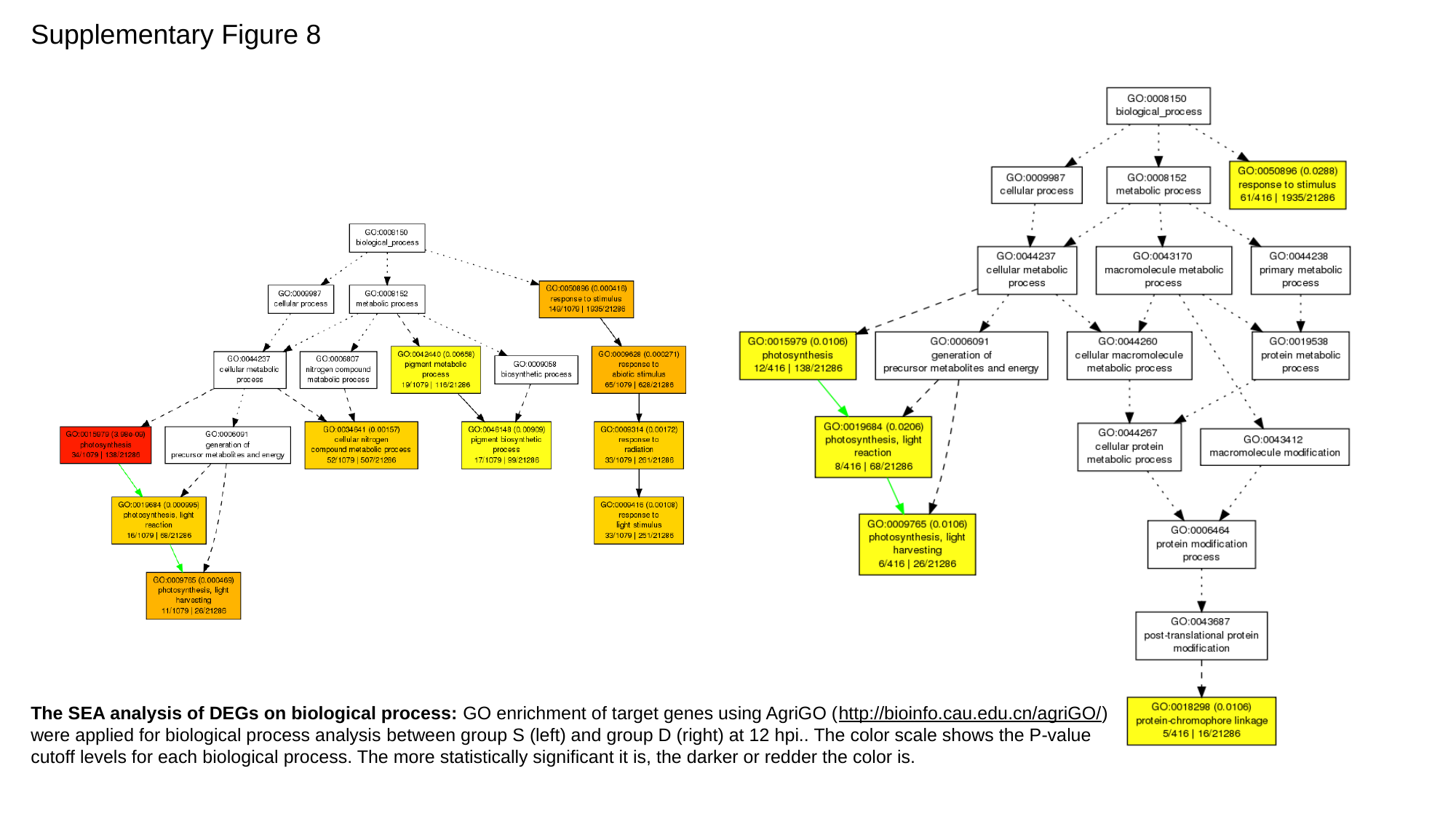

Supplementary Figure 8
The SEA analysis of DEGs on biological process: GO enrichment of target genes using AgriGO (http://bioinfo.cau.edu.cn/agriGO/) were applied for biological process analysis between group S (left) and group D (right) at 12 hpi.. The color scale shows the P-value cutoff levels for each biological process. The more statistically significant it is, the darker or redder the color is.

## Slide 9
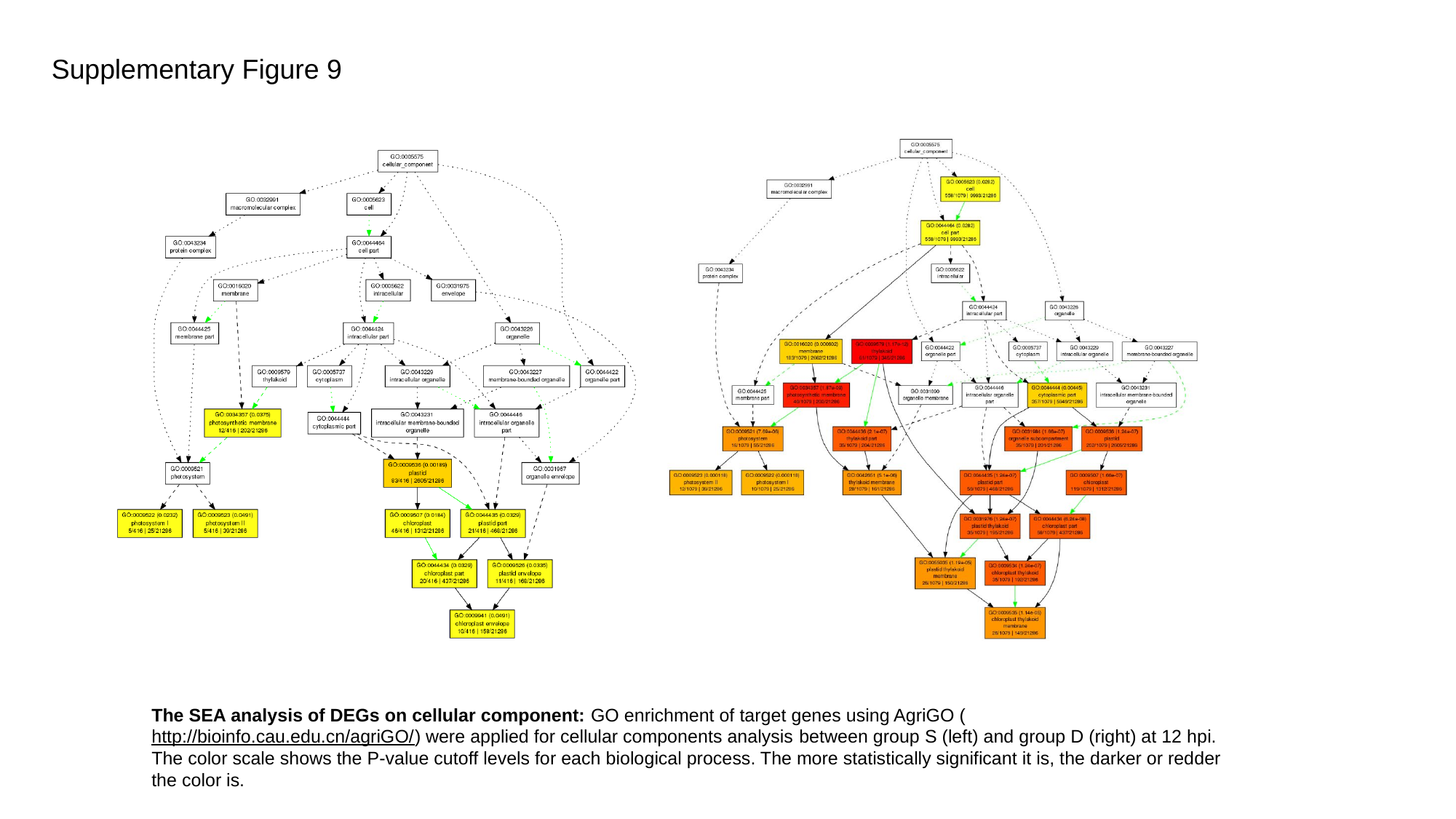

Supplementary Figure 9
The SEA analysis of DEGs on cellular component: GO enrichment of target genes using AgriGO (http://bioinfo.cau.edu.cn/agriGO/) were applied for cellular components analysis between group S (left) and group D (right) at 12 hpi. The color scale shows the P-value cutoff levels for each biological process. The more statistically significant it is, the darker or redder the color is.

## Slide 10
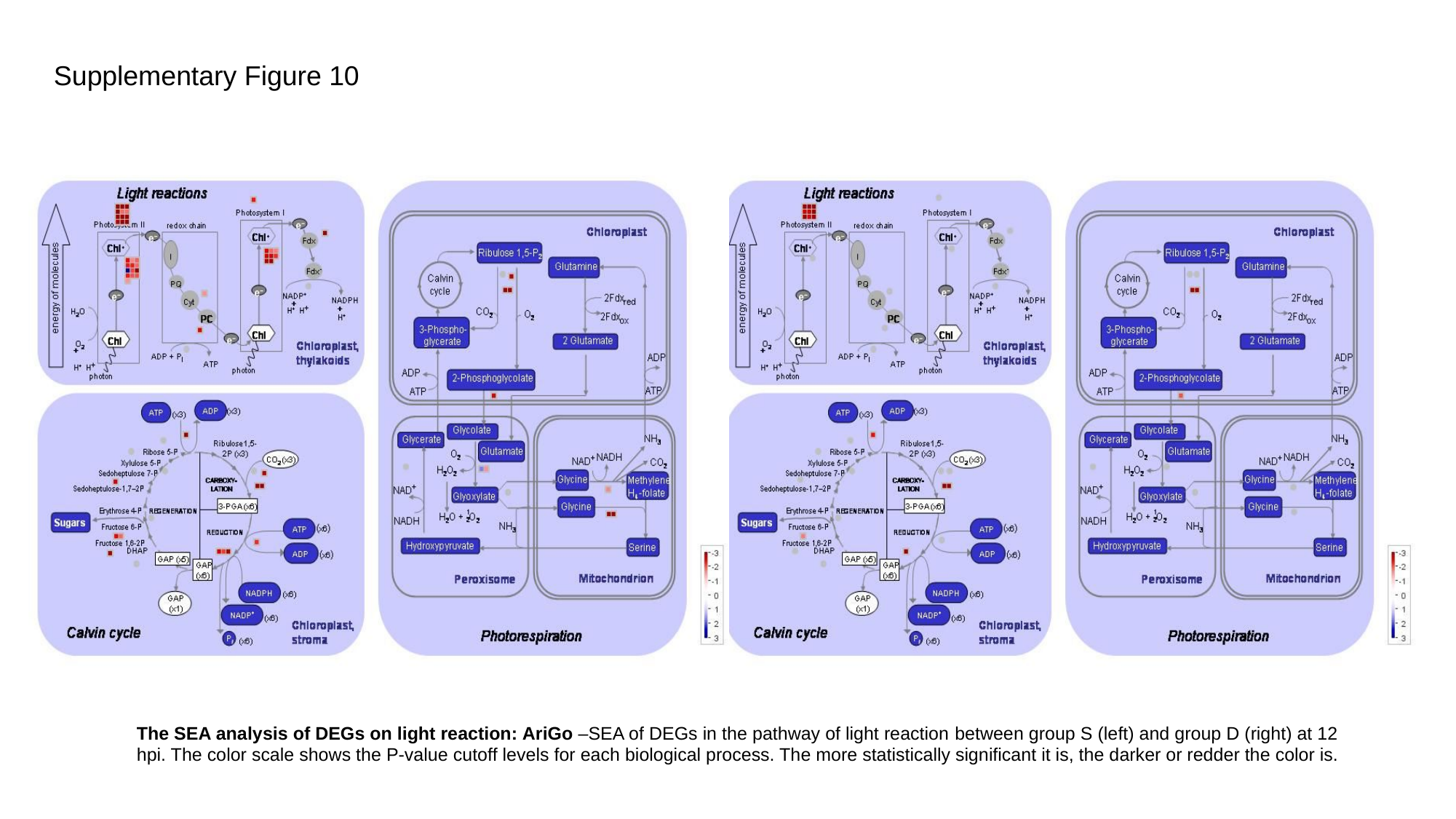

Supplementary Figure 10
The SEA analysis of DEGs on light reaction: AriGo –SEA of DEGs in the pathway of light reaction between group S (left) and group D (right) at 12 hpi. The color scale shows the P-value cutoff levels for each biological process. The more statistically significant it is, the darker or redder the color is.

## Slide 11
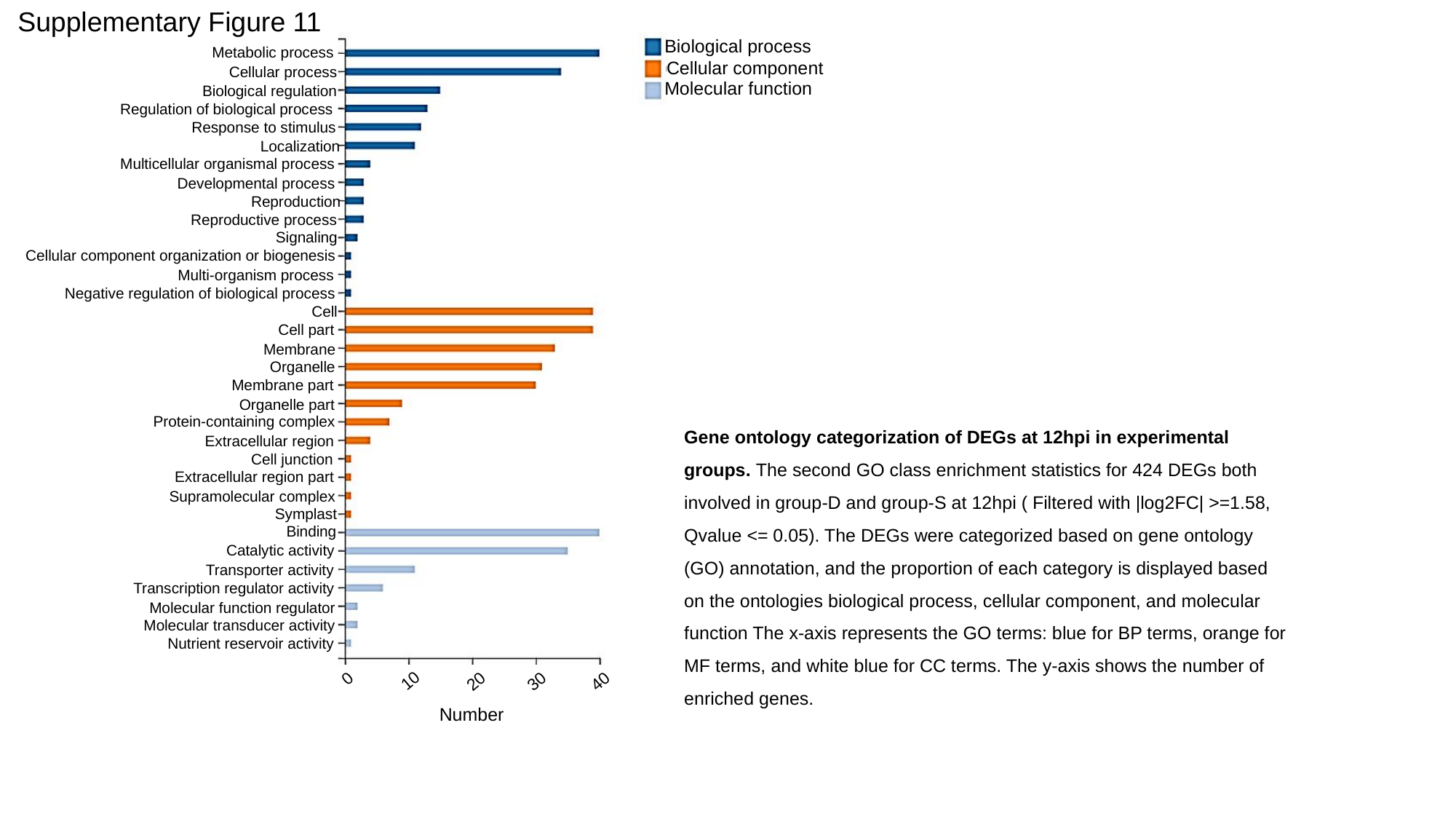

Supplementary Figure 11
Biological process
Metabolic process
Cellular component
Cellular process
Molecular function
Biological regulation
Regulation of biological process
Response to stimulus
Localization
Multicellular organismal process
Developmental process
Reproduction
Reproductive process
Signaling
Cellular component organization or biogenesis
Multi-organism process
Negative regulation of biological process
Cell
Cell part
Membrane
Organelle
Membrane part
Organelle part
Gene ontology categorization of DEGs at 12hpi in experimental groups. The second GO class enrichment statistics for 424 DEGs both involved in group-D and group-S at 12hpi ( Filtered with |log2FC| >=1.58, Qvalue <= 0.05). The DEGs were categorized based on gene ontology (GO) annotation, and the proportion of each category is displayed based on the ontologies biological process, cellular component, and molecular function The x-axis represents the GO terms: blue for BP terms, orange for MF terms, and white blue for CC terms. The y-axis shows the number of enriched genes.
Protein-containing complex
Extracellular region
Cell junction
Extracellular region part
Supramolecular complex
Symplast
Binding
Catalytic activity
Transporter activity
Transcription regulator activity
Molecular function regulator
Molecular transducer activity
Nutrient reservoir activity
0
10
20
30
40
Number

## Slide 12
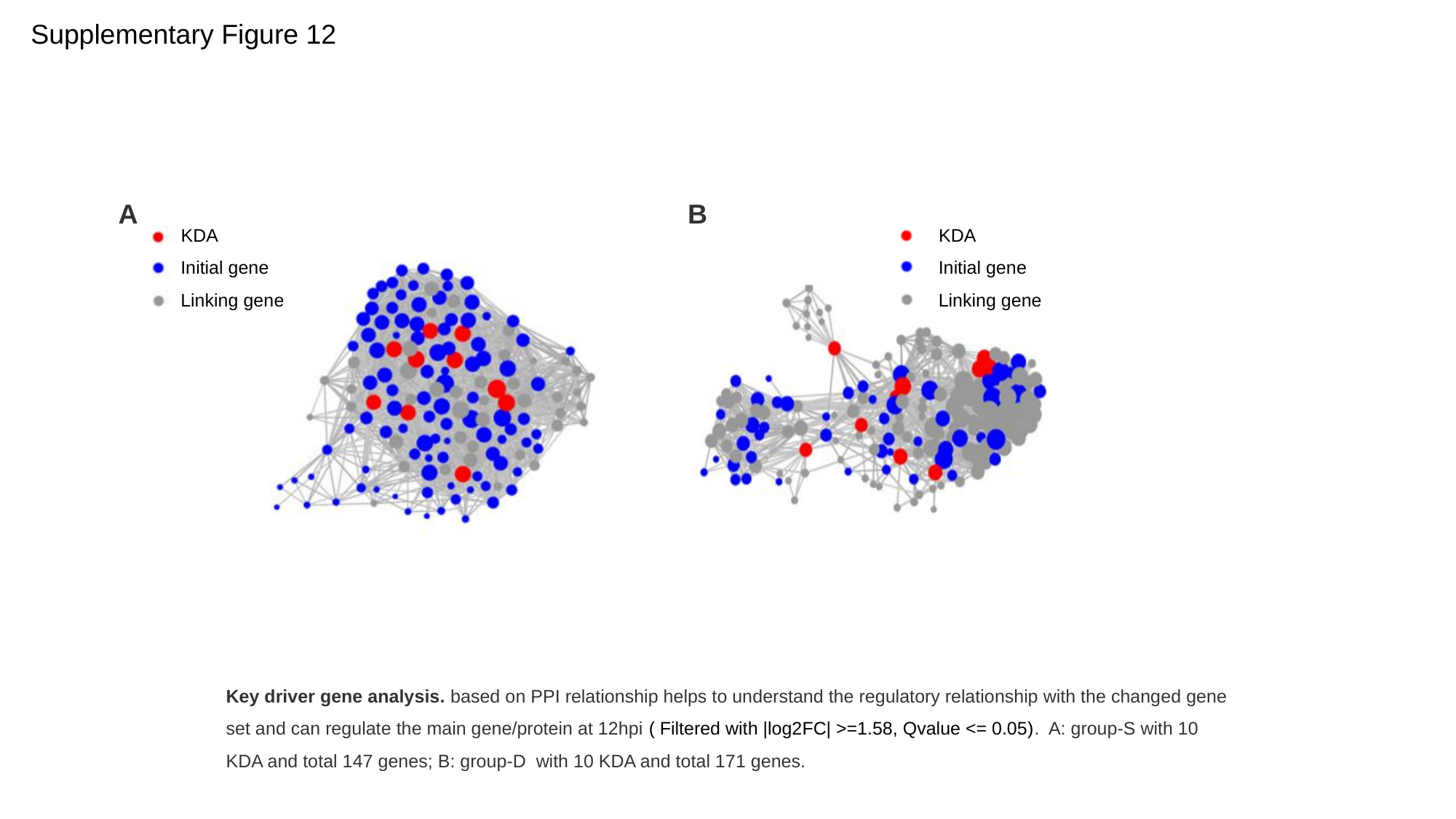

Supplementary Figure 12
 A
 B
KDA
KDA
Initial gene
Initial gene
Linking gene
Linking gene
Key driver gene analysis. based on PPI relationship helps to understand the regulatory relationship with the changed gene set and can regulate the main gene/protein at 12hpi ( Filtered with |log2FC| >=1.58, Qvalue <= 0.05). A: group-S with 10 KDA and total 147 genes; B: group-D with 10 KDA and total 171 genes.
